# Unraveling myriapod evolution: sealion, a novel quartet-based approach for evaluating phylogenetic uncertainty

**DOI:** 10.1093/nargab/lqaf018

**Published:** 2025-03-07

**Authors:** Patrick Kück, Mark Wilkinson, Juliane Romahn, Nathan I Seidel, Karen Meusemann, Johann W Wägele

**Affiliations:** Centre for Molecular Biodiversity Research, Leibniz Institute for the Analysis of Biodiversity Change, Adenauerallee 160, 53113 Bonn, Germany; Herpetology Lab, The Natural History Museum, Cromwell Road, London SW7 5BD, United Kingdom; Centre for Molecular Biodiversity Research, Leibniz Institute for the Analysis of Biodiversity Change, Adenauerallee 160, 53113 Bonn, Germany; LOEWE Centre for Translational Biodiversity Genomics (LOEWE-TBG), Senckenberganlage 25, 60325 Frankfurt am Main, Germany; Senckenberg Society for Nature Research, Senckenberganlage 25, 60325 Frankfurt am Main, Germany; Centre for Molecular Biodiversity Research, Leibniz Institute for the Analysis of Biodiversity Change, Adenauerallee 160, 53113 Bonn, Germany; Directorate, Leibniz Institute for the Analysis of Biodiversity Change, Adenauerallee 160, 53113 Bonn, Germany; Centre for Biodiversity Monitoring and Conservation Science, Leibniz Institute for the Analysis of Biodiversity Change, Adenauerallee 160, 53113 Bonn, Germany

## Abstract

Myriapods, a diverse group of terrestrial arthropods, comprise four main subgroups: Chilopoda (centipedes), Diplopoda (millipedes), Pauropoda, and Symphyla. Recent phylogenomic studies affirm Myriapoda’s monophyly and the monophyletic status of each subgroup but differ in their relationships. To investigate these relationships further, we reanalyzed a transcriptomic dataset of 59 species across 292 single-copy protein-coding genes. Departing from conventional methods, we employed a novel approach that relies on information from polarized quartets (i.e., sets of four orthologous sequences, with one being an outgroup) to evaluate molecular phylogenies. This Hennigian analysis reduces misleading phylogenetic signals in molecular data caused by convergence, plesiomorphy, and rate heterogeneity across sites and across lineages. Our findings reveal that some species, especially those with long root-to-tip distances, disproportionately contribute misleading signals. Analyses using conventional likelihood-based phylogenetic methods suggest that Chilopoda and Diplopoda are sister taxa. By contrast, analyses incorporating novel filters designed to minimize conflict among phylogenetically confounding signals support the monophyly of Progoneata, aligning with morphological evidence. Simulations validate the reliability of our approach, demonstrating its potential to resolve myriapod evolutionary relationships and highlight uncertainty.

## Graphical Abstract

**Graphical Abstract:**
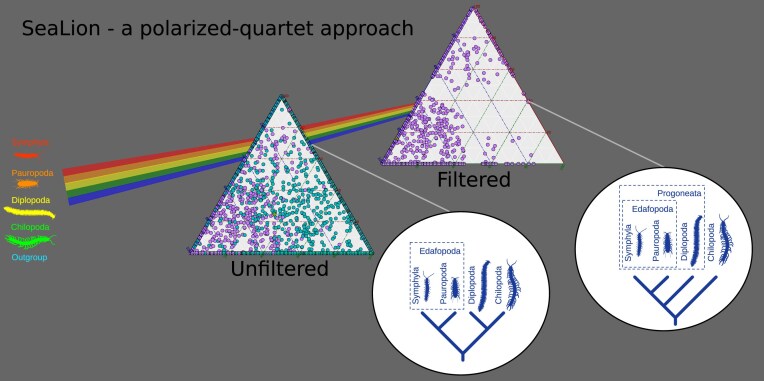

## Introduction

**Figure 1.**
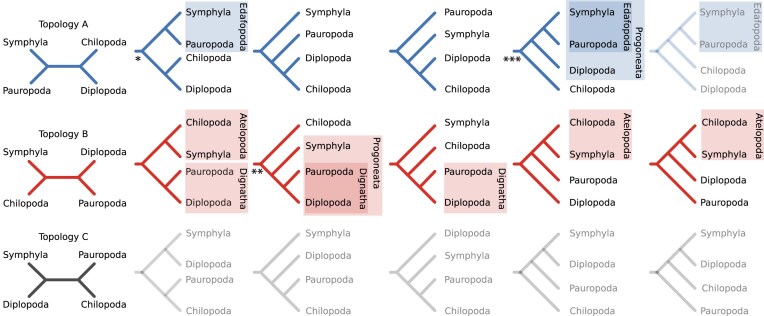
Proposed hypotheses regarding the relationships among the major myriapod lineages: Chilopoda, Diplopoda, Symphyla, and Pauropoda. Unrooted quartet topology A (depicted in blue, left) suggests a grouping of Pauropoda + Symphyla and Chilopoda + Diplopoda. The row shows various trees derived from this topology through different rootings. * denotes the best ML tree from Szucsich et al. [3]. Unrooted topology B (depicted in red) proposes a grouping of Chilopoda + Symphyla and Diplopoda + Pauropoda, with the column displaying diverse trees resulting from various rootings. ** indicates the primary ML tree inferred by Fernandez et al. [2], resulting in Progoneata with monophyletic Dignatha. *** The relationship inferred by Regier et al. [4] not only supports the Progoneata hypothesis but also suggests the sister-group relationship of Symphyla and Pauropoda, known as Edafopoda [5]. However, it is worth noting that this relationship is incompatible with the Dignatha clade. Topology C (depicted in gray) posits Diplopoda + Symphyla and Chilopoda + Pauropoda, with the row exhibiting trees derived from different rootings. However, none of them is supported by existing studies [3].

Myriapods, a diverse group of terrestrial arthropods with approximately 15,000 known extant species, has four major subgroups: the species-rich Chilopoda (centipedes) and Diplopoda (millipedes), along with the less species-rich Pauropoda and Symphyla [1]. Recent phylogenomic studies support the monophyly of Myriapoda and of each major subgroup [2, 3] but differ in the relationships among these subgroups (Fig. 1).

The first published phylogenomic study of myriapod subgroup relationships, conducted by Fernandez et al. [2], corroborated trees inferred from morphological data, supporting a sister-group relationship of Diplopoda and Pauropoda, known as Dignatha [6, 7]. This millipede-pauropod group shares modified mouthparts that lack a separate first maxilla, the second maxillae are fused (Gnathochilarium), and the first trunk segment has no appendages (collum segment). Symphyla were proposed to be the closest relatives of Dignatha, which lent support to the concept of a monophyletic group known as Progoneata (encompassing Diplopoda, Pauropoda, and Symphyla). This proposed relationship was founded primarily on the unusual proximity (among arthropods) of their genital apertures to the anterior end of the trunk [8]. The Fernandez et al. [2] tree is equivalent to an unrooted quartet topology that groups Diplopoda with Pauropoda and Chilopoda with Symphyla. Among all the published molecular phylogenies, only the trees presented in Regier et al. [9] and Rehm et al. [10] also include this topology.

By contrast, some investigations of morphological and molecular data have supported a sister-group relationship between Symphyla and Pauropoda, for example [11–13]. Notably, the phylogenomic study by Regier et al. [4] not only confirmed the monophyletic grouping of Progoneata but also supported the sister-group relationship of Symphyla and Pauropoda, referred to as Edafopoda [5], which is incompatible with Dignatha. Miyazawa et al. [14] presented an additional molecular phylogenetic tree, which supports the idea of a sister-group relationship between Chilopoda and Diplopoda, with Symphyla arising from the basal split.

A more recent phylogenetic analysis of transcriptomic data [3] also contradicted the tree proposed by Fernandez et al. [2] and the insights derived from morphological evidence (for an in-depth morphological review, see Edgecombe [8]). The inconsistency lies both in the topology and in the position of the root. Szucsich et al. [3] identified a strong and consistent signal for a topology, where Pauropoda and Symphyla (Edafopoda) are the sister-group of Chilopoda and Diplopoda. This phylogenetic arrangement, which places Chilopoda and Diplopoda as sister taxa, corresponds to the conclusions of Dong et al. [11], Gai et al. [12], Miyazawa et al. [14], Podsiadlowski et al. [13], Regier et al. [4], and Zwickl et al. [5]. This relationship has been consistently recovered using various data types, including amino acid and nucleotide sequences. Furthermore, this phylogenetic topology has been validated through methods such as Four-cluster Likelihood-Mapping [15] and the approximately unbiased test (AU-test: [16]).

However, it is important to note that the position of the root is sensitive to the selection of outgroup sequences. To investigate the potential influence of outgroup selection, Szucsich et al. [3] explored the matter using either exclusively chelicerates and onychophorans as the outgroup or solely pancrustaceans. Although most of these analyses support a sister-group relationship between Pauropoda and Symphyla, the placement of Chilopoda as the sister-group to Diplopoda was not always confirmed. While Szucsich et al. [3] suggested that this relationship “appears likely,” it is worth noting that the possibility of Diplopoda being the sister-group to Edafopoda, supporting the Progoneata clade, was also not rejected statistically [3].

Conflicting signals exist in phylogenetic data when site patterns support mutually incompatible bipartitions (i.e., groups that cannot be displayed on the same tree). There are various sources of conflicting signals, such as incomplete lineage sorting (ILS), recombination, or reticulation events like introgression, hybridization, and horizontal gene transfer [17–19]. Frequently, conflicting signals result from substitutions that produce similarities between unrelated lineages, leading to both stochastic and systematic errors in inferred phylogenetic trees. Systematic errors can arise when the amount of data is large enough to reduce stochastic errors to negligible levels, yet model violations introduce biases that skew tree estimations.

Deep divergences may be challenging to resolve with confidence in phylogenetic analyses due to the decay of the phylogenetic signal over time [20] and rapid radiations, where short branches provide limited time for informative changes to accumulate [21, 22]. Additionally, systematic biases such as compositional heterogeneity and long-branch attraction (LBA), which result from model violations, remain persistent challenges in phylogenetic inference [20, 23–27].

Interactions among multiple confounding factors, such as phylogenetic signal, compositional biases, and rate heterogeneity, can significantly complicate the process of phylogenetic inference. For example, compositional heterogeneity among sequences can bias phylogenetic estimates significantly, and even small differences in this heterogeneity may lead to incorrect tree topologies [20, 27, 28]. These challenges underscore the importance of addressing both stochastic and systematic biases in molecular phylogenetic data.

Although maximum likelihood (ML) and Bayesian inference (BI) methods tend to be robust to model violations, variations in rates of sequence evolution across a tree can introduce significant systematic biases that undermine the performance of these methods [20, 23, 25, 29–38]. When substitution models fail to adequately account for variations in substitution rates, compositional heterogeneities, character state polarity, or the erosion of phylogenetic signal, they can lead to erroneous phylogenetic inferences that appear to be well-supported by misleadingly high bootstrap proportions or posterior probabilities [23, 24, 32, 37, 39–44]. Rate variation can be further divided into site-specific and lineage-specific rate variation, both of which can interfere with phylogenetic reconstruction when not properly accounted for. Therefore it is crucial to carefully evaluate signals in the data, assess the fit of the data to inferred phylogenetic trees, and ensure the adequacy of the data for accurate phylogenetic analysis.

Identifying, partitioning and reducing conflicting signal in phylogenomic datasets by appropriate filtering approaches that address estimation errors is helpful for understanding and potentially resolving difficult phylogenetic questions. Here we introduce SeaLion, a new PhyQuart-based phylogenetic analysis pipeline [45] for dissecting and evaluating signals supporting alternative tree topologies, and use it to identify conflicting signals within the “STRICT” nucleotide dataset from Szucsich et al. [3].

Quartet approaches break down large phylogenetic problems into smaller, more manageable ones, avoiding the rapid expansion of tree-space as the number of taxa increases. The information from individual quartet problems is then used to reconstruct a full phylogenetic tree through a process of quartet or supertree amalgamation [46–48]. This “divide and conquer” strategy can help to isolate or partition biases, potentially freeing some quartets from these issues altogether [46, 49–52]. Quartet-based supertree methods are particularly appealing for large-scale phylogenetic reconstructions, aiming to identify the tree(s) that best reflect the information in individual quartet trees (e.g. [53–55]).

PhyQuart evaluates the support for different tree topologies based on site-patterns in sequence alignments. By applying Hennigian logic [56, 57], this approach aims to avoid errors caused by symplesiomorphies (shared ancestral traits) and integrates corrections based on ML estimates [43, 45], offering an alternative to traditional phylogenetic methods.

SeaLion extends the capabilities of PhyQuart by integration into a comprehensive tree reconstruction and evaluation framework. This allows for precise analyses of multiple quartets, combining their evaluations to distinguish between well-supported phylogenetic hypotheses and those for which there is substantial conflict in the data. Unlike other methods that assess confidence across entire trees, SeaLion tackles specific phylogenetic questions by focusing on relationships among sets of predefined groups (such as myriapod subgroups) that are assumed to be monophyletic. This approach is particularly designed for analyzing complex scenarios involving a mix of short and long internal branches between predefined clades, each of which includes one or many species.

Additionally, SeaLion aims to enhance the accuracy of phylogenetic inference by filtering out conflicting signals within quartets, such as random noise and plesiomorphic character similarities. Noise refers to random or misleading patterns in the data that do not reflect any evolutionary relationship, while plesiomorphic character similarities are genuine homologies shared among taxa due to inheritance from a common ancestor but are not informative for resolving more derived evolutionary relationships. By addressing these factors, SeaLion prioritizes patterns that more reliably reflect true evolutionary relationships. SeaLion uses objective functions combined with an hill-climbing algorithm to implement two distinct filters, which can be applied either individually or in combination. The “RISK” filter evaluates the balance between potential shared derived characters and convergent signals among quartets, while the “DIST” filter measures the overall conflict between quartet trees to identify the quartets with the least conflict (for more details, see “Materials and methods” and Supplementary File S1).

This new approach may address challenging relationships and highlight limitations in the dataset’s ability to resolve certain relationships.

## Materials and methods

### Phylogenomic data

The dataset employed in our analysis, derived from Szucsich et al. [3], is constructed from transcriptome data from 59 panarthropod species with a strict gene coverage criterion, encompassing 292 single-copy protein-coding genes per species. In our analysis, we focused on the second-codon-only dataset, with a length of 95,797 aligned sites on second-codon nucleotide level (overall information content (IC): 0.30, alignment completeness score 82.53%; see Szucsich et al. [3]). As outlined in the original study [3], this dataset displayed the lowest violation of stationary, reversible, and homogeneous (SRH) conditions, thereby exhibiting reduced heterogeneity across lineages. The 59 species include representatives of each of the four myriapod subgroups (30 species), as well as outgroup taxa, namely Pancrustacea, Chelicerata, and Onychophora (29 species).

### Myriapod clades without Edafopoda

Our analysis is based on the classification of the included species into four putatively monophyletic myriapod subgroups (ingroup clades) while assigning any remaining species to the outgroup. This classification follows the principle that the outgroup does not need to be monophyletic and may include taxa from other arthropod lineages, such as onychophores, which are considered protarthropods. These taxa provide valuable context by potentially retaining ancestral character states that have evolved differently in Myriapoda. However, we acknowledge that molecular evolution can vary significantly among genes, with some sites evolving more slowly and retaining conserved states over extended periods.

Thus, while the outgroup contributes insights into ancestral character states and is particularly useful for rooting topologies and determining branch polarity, the interpretation of plesiomorphic versus apomorphic character states must account for the limitations of quartet-based analysis. Specifically, while our method is designed to highlight and minimize noise and reduce systematic bias through averaging and filtering, it is not fully immune to errors stemming from extreme deviations from model assumptions.

Relationships within these assumed clades are not the focus of this analysis; resolving them would require separate investigations targeting subgroup relationships. This focus on higher-level topology ensures that our approach effectively evaluates broader evolutionary patterns while maintaining a clear framework for addressing inherent complexities in the data.

We differentiate between clade-quartets and species-quartets in our analysis. Clade-quartets represent broader phylogenetic hypotheses among assumed monophyletic groups that we are testing. In contrast, species-quartets include one representative species from each group in the corresponding clade quartet and are used for site-pattern analyses. These analyses help to distinguish between phylogenetic signal and noise. By “phylogenetic signal,” we refer to patterns in the data that reflect true evolutionary relationships. Conversely, “noise” encompasses random or misleading patterns, such as those arising from sequencing errors, substitutional saturation, or homoplasy. A specific focus is placed on identifying plesiomorphic signals (character states shared due to inheritance from a common ancestor) that may mislead phylogenetic inference by being mistaken for evidence of monophyly when they are not.

**Figure 2.**
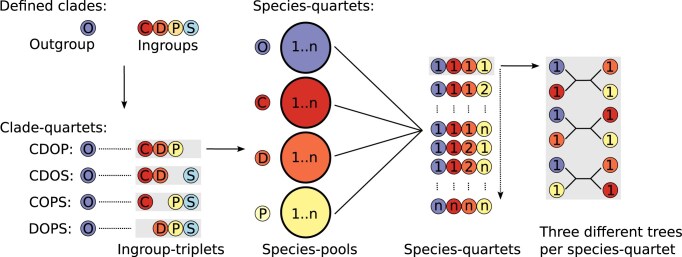
Schematic overview of quartet construction and tree analysis. For a set of defined outgroup and ingroup clades (top left), all possible clade-quartets are formed by combining each ingroup triplet with the outgroup (bottom left). For each clade-quartet, all possible species-quartets are generated by selecting one species from each clade’s species pool (species 1...n), ensuring each quartet forms a unique species combination (“1111” to “nnnn”), with three different tree topologies to be analyzed.

In this study, we analyse four different sets of clade-quartets, each with a corresponding set of species-quartets that feature one outgroup species (O) and one species from three of the four myriapod ingroup clades (Symphyla: S, Chilopoda: C, Pauropoda: P, and Diplopoda: D). For each of these four clade-quartet combinations (CDOS, CDOP, COPS, DOPS), three possible outgroup-rooted tree topologies are evaluated (Fig. 2). A summary of the number of species assigned to each clade is provided in Table 1.

**Table 1.** Summary of species numbers (N) assigned to myriapod clades and the outgroup^1^

Clades without Edafopoda	Code	N Species		
Chilopoda	C	16		
Diplopoda	D	10		
Pauropoda	P	1		
Symphyla	S	3		
Outgroup	O	29		
**Clade-quartet**	CDOS	CDOP	COPS	DOPS
**Clades with Edafopoda**	**Code**	**N Species**		
Chilopoda	C	16		
Diplopoda	D	10		
Edafopoda (Pauropoda+Symphyla)	X	4		
Outgroup	O	29		
**Clade-quartet**	CDOX			

^1^Four myriapod clades are considered and in addition an outgroup with different panarthropod species. Where the Edafopoda are used as potential monophylum, species of Pauropoda and Symphyla form a single group. “Code” are the abbreviations for clades used in text and figures.

**Figure 3.**
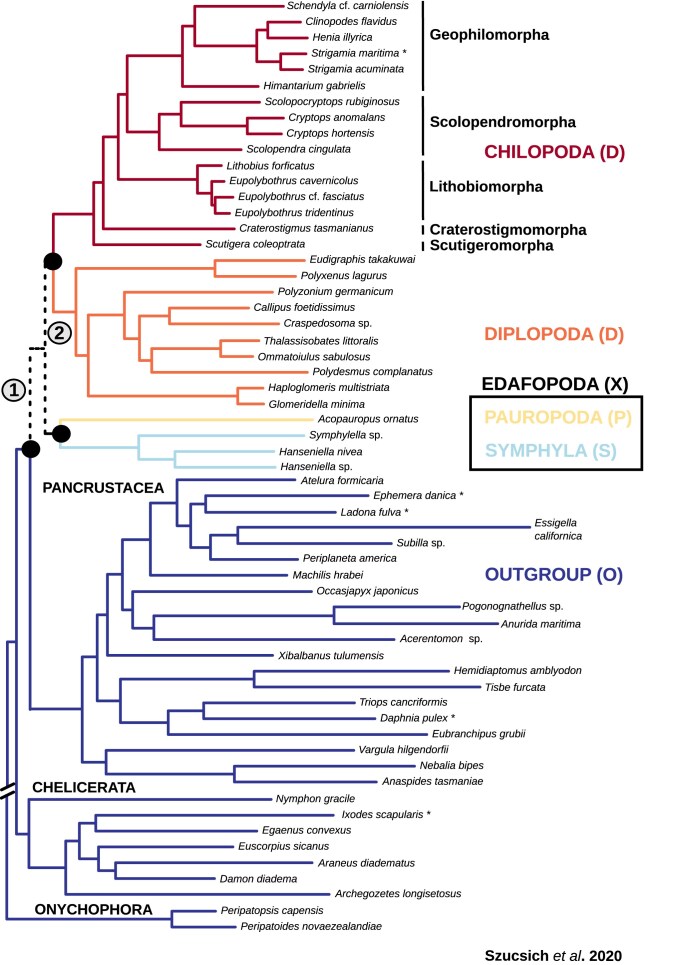
Tree topology from Szucsich et al. [3] with groupings relevant for the present study. The four myriapod subgroups are connected by the internal branch (2). The root for Myriapoda is defined by adding outgroup taxa (branch 1). While our analysis of five groups (myriapod subgroups plus outgroup) focuses on both internal branches, the more streamlined four-clade analysis combines Pauropoda and Symphyla into a clade called Edafopoda (X) and concentrates solely on the outgroup’s position relative to the remaining myriapod clades.

Our clade classification facilitates analyses of two specific internal branch relationships within the original ML tree as presented in Szucsich et al. [3]. These relationships pertain to the internal branching among the four myriapod subgroups and the determination of the position of the root (Fig. 3).

### Myriapod clades with Edafopoda

Building upon the hypotheses proposed by Szucsich et al. [3], we extended our analyses to include topologies incorporating the hypothetical clade Edafopoda (Symphyla + Pauropoda), which reduces the number of ingroup clades to three and results in a distinct composition of species-quartets, differing from the previous clade analysis. The analysis specifically concentrates on potential outgroup (root) positions among the internal branches combining the three ingroup clades. Thus, we wanted to test how close the Diplopoda are to Chilopoda.

### SeaLion processing

SeaLion is specifically designed for the reconstruction of rooted trees using clades as terminal taxa. The overlap of some clades across different quartet trees allows for the assembly of more comprehensive trees that represent all defined clades, referred to here as rooted clade-trees.

**Figure 4.**
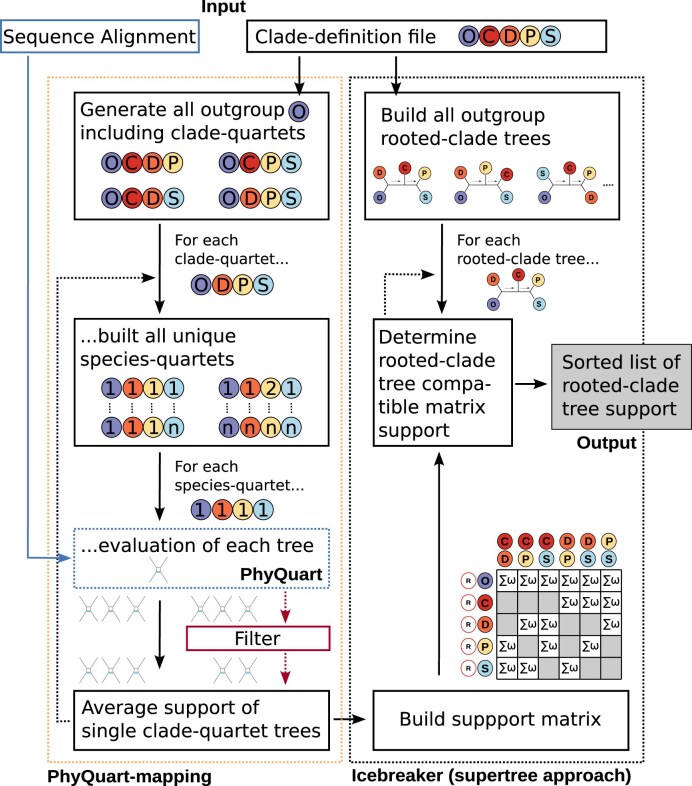
Shematic overview of SeaLion process steps. SeaLion reads individually defined clades and their assigned sequences from the clade-definition file. In the PhyQuart-mapping process (dashed yellow box on the left), SeaLion generates all possible clade-quartets, including the outgroup. It then analyzes the associated species-quartets for split-patterns in quartet-based sequences, drawn from the original alignment, using the PhyQuart algorithm (dashed blue box). After analyzing all species-quartets for a particular clade combination, the species-quartets are optionally filtered based on reduced tree signal (red box). In the Icebreaker process (dashed gray box on the right), the median support of species-quartets inferred for individual clade-quartet trees (ω) is then translated into a pairwise-compatibility support matrix (bottom). Following this, rooted-clade trees are constructed from the complete set of defined clades, and tree corresponding (unfiltered or filtered) summed-up matrix support is used as final score for each tree. At the conclusion of the supertree (Icebreaker) process, SeaLion generates for each (unfiltered or filtered) analysis, among other outputs, a list of all rooted-clade trees sorted by their final scores.

In practice, the SeaLion pipeline consists of three consecutive steps: (i) the analysis of individual species-quartets, including the outgroup, using the PhyQuart algorithm, ensuring no more than one species per clade; (ii) the aggregation of relevant species-quartet PhyQuart scores for each clade-quartet tree, using the median value of the PhyQuart scores; and (iii) the inference of relationships among more than four predefined clades, relying solely on the support from larger rooted-clade trees derived from clade-quartet analyses, employing a new quartet-based supertree algorithm. For a schematic overview of all three process steps, refer to Fig. 4, and for a more detailed overview, see Sections 1.1–1.3 in Supplementary File S1.

SeaLion uses the median of PhyQuart scores as the measure of aggregate clade-quartet support because it is less sensitive to outliers than the mean, making it more reliable for skewed or non-normally distributed data. The median provides a more accurate central value and is easier to interpret, especially when analyzing ordinal or categorical data, making it well-suited for assessing clade-quartet support in our analysis.

The new quartet-based supertree approach (Icebreaker) summarizes support for all possible tree-based relationships among clades in a pairwise-compatibility support matrix (Fig. 4). From this matrix, support for partitions of predefined clades among the rooted trees of clades are derived to assess compatibility. The approach identifies the rooted topology of clades with the highest maximum-clique support, representing the most compatible relationships among clades (see Section 1.3 in Supplementary File S1).

For example, a rooted tree of the clades (O,((C,D),(P,S))) is maximum-clique compatible, represented by four clade-quartet trees: (O,(P,(C,D)), O,(S,(C,D))), (O,(C,(P,S))), and (O,(D,(P,S))). In this case, the maximum clique is the largest group in a graph based on the compatibility matrix, where all combinations of rooted and derived clade pairs are pairwise compatible.

The evaluation of support for partitions of predefined clades for all possible trees containing more than four predefined clades bypasses the maximum-quartet-consisteny problem [58] and avoids reliance upon a heuristic for quartet or supertree amalgamation [46–48].

#### Single species-quartet analysis

The number of individual quartets varies across each clade combination in our analyses, ranging from 870 to 13,920 (Table 2). In each quartet analysis, the PhyQuart algorithm considers the Hennigian distinction between newly evolved characters unique to a monophylum (apomorphic characters) and older characters also occurring elsewhere (plesiomorphies). This distinction is crucial for identifying clade relationships. PhyQuart was specifically designed to handle polarized character states in rooted trees, allowing for a clear distinction between older plesiomorphic states and more recently evolved apomorphic ones [45]. The use of rooted trees provides an additional advantage by helping to address issues associated with polarization and evolutionary directionality, ensuring a more accurate representation of evolutionary relationships. The outgroup determines the root position in each quartet, and this rooting is essential for the correct interpretation of branch polarization, from the root to the terminal leaves. This polarization is critical for filtering out irrelevant branch support, particularly for plesiomorphic homologies, as detailed in Kück and Wägele [43]. By utilizing PhyQuart scores as the optimality criterion, a topology is suggested for all possible tree arrangements rooted with the pre-defined outgroup.

**Table 2.** The total number of individual quartet analyses (Nq) is presented for rooted species-quartets, encompassing both unfiltered and filtered datasets using the “RISK” filter alone and in combination with the “DIST” filter, along with the corresponding optimized threshold values (“L_RISK_,” “L_DIST_”; see Section 1.4.3 in Supplementary File S1.). The number of species-quartets results from the combination of species considered in each clade (Table 1). Note: CDOX combines Symphyla and Pauropoda into Edafopoda (“X”), leading to a single four-clade analysis. All other clade-quartets are part of the broader myriapod analysis with Symphyla and Pauropoda defined as own clades

	Nq “Unfiltered”	L_RISK_	Nq “RISK”	L_DIST_	Nq “RISK+DIST”
CDOP	4.640	0.77	1.495	0.1	1.288
CDOS	13.920	0.77	3.780	0.1	3.388
COPS	1.392	0.76	507	0.1	492
DOPS	870	0.76	217	0.1	209
CDOX	18.560	0.77	5.275	0.1	4.676

For each quartet of species, only alignment sites devoid of missing or ambiguous character states are considered. This approach ensures the elimination of uncertainties, although it also implies that the set of sites considered can vary slightly for each quartet.

To estimate the expected number of potentially convergently evolved chance similarities contributing to a potential false phylogenetic signal (Nc), we employ ML inference implemented in the P4 package by Foster [28], as described in Kück et al. [45]. For each of the three tree possibilities, P4 estimates the mean number of tree-supporting site patterns derived from the other two trees. Assuming that one of the other two trees is correct, patterns supporting the original tree under investigation would be regarded as false signal. This approach facilitates the optimization of both branch lengths and model parameters across the five branches of the quartet tree (internal and terminal branches), with optimization based on the original quartet alignment. This flexibility enables each branch to evolve under potentially distinct evolutionary processes. To be considered a reliable signal, the number of supporting sites (Na), which directly reflects the phylogenetic signal identified from the quartet alignment, must exceed the anticipated number of chance similarities (Na > Nc). Both values (Nc and Na) are specific to each of the three species-quartet trees under consideration.

The support values are determined by the respective Nc/Na ratio, where Nc represents conflicting sites (potential noise or misleading signals), and Na represents supporting sites (reflecting the potential phylogenetic signal). The lower the Nc/Na ratio, the stronger the potential for phylogenetic signal in the quartet-tree under investigation. These support values are then normalized to produce relative support measures. Specifically, each tree’s support score (Si) is divided by the sum of the scores across all three possible trees, as shown below:


\begin{equation*} R_i = \frac{S_i}{S_1 + S_2 + S_3}, \quad \text{for } i \in \lbrace 1, 2, 3\rbrace \end{equation*}

where R_i_ denotes the normalized support for tree i. The normalized support values, which range from 0 to 1, represent the relative signal in favor of a given tree topology among the three possible alternatives for a quartet of sequences within each rooted tree of clades. A value of 1 indicates that all supporting sites favor the given tree, while 0 represents no sites supporting the tree. A high support value (e.g., 0.8) suggests a stronger relative signal for the given topology compared to the alternatives. However, it does not imply the absence of conflicting data. Instead, the value reflects the balance of conflicting and supporting sites after normalization. For instance, a value of 0.8 indicates that 80% of the total normalized support is attributed to the best tree, with the remaining 20% distributed across the other two topologies.

In cases of extreme conflict, the best and second-best trees may each have normalized support values close to 0.5, reflecting an almost equal distribution of signal among conflicting sites. Conversely, when the normalized support value for the best tree exceeds 0.8, it indicates a relatively strong difference in signal favoring the best tree. This approach provides a clear framework for interpreting the strength of support and the level of conflict in the data.

#### Single clade-quartet analysis of multiple species-quartets

As the pipeline utilizes sequences from individual species to calculate scores for clade-quartets, PhyQuart values are obtained for all combinations of sequences within a clade combination. Consequently, for each clade-quartet, the PhyQuart algorithm evaluates each tree, and the scores are averaged by computing the arithmetic median from quartet topologies of individual species within the tree (see Section 1.2 in Supplementary File S1).

This process aims to identify the optimal topology for the given quartet combination of clades. In this context, high scores indicate robust support and a strong signal for a topology, while low scores suggest significant data conflict and the potential for systematic bias arising from branch length heterogeneity. Such bias may result from effects like branch attraction due to convergent character states or shared plesiomorphic character states [43]. In conventional phylogenetic analyses, these factors can lead to misleadingly high support values for incorrect topologies.

#### Larger tree analysis of multiple clade-quartets

In the following step, the result is used to assess the relationships among more than four clades (see Section 1.3 in Supplementary File S1). This evaluation entails the assessment of every potential tree for the specified group of clades. Following this analysis, SeaLion generates a compilation of tree individual quartet support values for each of the top 100 (as per the default setting) rooted clade relationship.

#### Filtering species-quartets

While it is possible to conduct analyses with all species-quartets of a clade combination, certain species-quartets may lack a dominant topology due to the presence of conflicting phylogenetic signals. In such cases, where potentially convergent characters cannot be distinguished from homologous character states, these species-quartets with no dominant topology can be excluded from the final tree assembly. This exclusion is facilitated by two filters provided by SeaLion: the “DIST” filter and the “RISK” filter.

The “DIST” filter evaluates support distances between topologies within a species-quartet by comparing the difference in PhyQuart scores between the top-ranked (QT_best_) and the second-best topology (QT_second_). This approach assesses the level of conflict between the two topologies. A strong phylogenetic signal is expected to produce a pronounced support difference, whereas smaller differences may indicate weak or misleading signals. The filter quantifies this conflict using a support distance metric (SD_1, 2_), defined as the difference between the support values of QT_best_ and QT_second_.


\begin{equation*} SD_{1,2} = \text{Support } QT_{best} - \text{Support } QT_{second} \end{equation*}

Species-quartets with SD_1, 2_ below a threshold (L_DIST_) are excluded from the analysis, as such quartets likely lack a dominant phylogenetic signal. Conversely, quartets with SD_1, 2_ above the threshold are retained, as the signal conflict between QT_best_ and QT_second_ is minimal.

The “RISK” filter evaluates the ratio of convergent signal (Nc) to apomorph signal (Na) for each of the three possible topologies of a species-quartet. The Nc/Na ratio helps identify species-quartets where the signal supporting a particular tree is strong and distinct from potential noise or convergence. A lower Nc/Na ratio value indicates that the apomorph signal dominates, supporting the selected topology. Conversely, a higher Nc/Na ratio suggests that the convergent signal overwhelms the phylogenetic information, warranting the quartet’s exclusion.

The design of the “RISK” filter adheres to three guiding principles: (i) conflict minimization: the larger the difference in signal strength between the best-supported tree and the two alternative topologies, the lower the conflict, and the more reliable the best-supported tree is considered; (ii) signal dominance: a Nc/Na ratio closer to 0 for the best-supported tree indicates a lower influence of convergent signal (Nc), making the signal more robust; and (iii) risk assessment: When the Nc/Na ratio is closer to 1, the best-supported tree is more susceptible to the influence of convergent signals, increasing the likelihood of an incorrect tree being favored.

To determine whether a species-quartet is retained or rejected, the Nc/Na ratio of the best-supported tree is compared to a threshold (L_RISK_). If the Nc/Na ratio is below this threshold, the quartet is considered reliable and included in further analysis. Otherwise, it is excluded, as the phylogenetic signal may be unreliable.

Threshold optimization for both filters, “DIST” and “RISK,” is performed using a specialized uphill-climbing algorithm integrated into SeaLion, ensuring robust performance across datasets. These filters work independently or in combination to refine the input data by excluding quartets with ambiguous or misleading signals.

We employed SeaLion to analyze the nucleotide dataset of Szucsich et al. [3] in three ways: without applying any filters, using the “DIST” filter alone, and combining the “RISK” and “DIST” filters in the same analysis. For further details, including formulaic descriptions and theoretical background, please refer to Section 1.4 in Supplementary File S1.

#### Output data

At each stage of the analysis, SeaLion allows a detailed evaluation of the potential for deceptive signals that might mislead phylogenetic inferences, providing a nuanced evaluation of the critical balance between support and conflict for phylogenetic hypotheses.

In the current analysis, we generated ternary plots for individual quartets analyzed using PhyQuart. In these plots, the position of points relative to the corners illustrates the identified signal strength of each species-quartet in relation to the three possible clade-quartet topologies. SeaLion allows the printing of additional plots and text files that provide insights into the individual quartet- and median-related clade-quartet tree-likeness of the data (see manual of SeaLion).

### Data simulations

To assess the reliability of SeaLion, we performed some simulations within the parameter space of the original ML tree using Indelible [59]. All simulations are based on the original number of site positions, estimated branch lengths, and substitution model parameters (see Szucsich et al. [3]). We used 20 data sets generated on the original ML tree as described in Szucsich et al. [3], with Chilopoda and Diplopoda in a sister-group relationship, as well as 20 data sets generated on an alternative tree topology with the clade Progoneata. For the simulation test, these would be the correct trees that should be recovered with our method. Variations of data sets were used (ten data sets with and without indel events for each of the two trees, respectively). In order to achieve a distribution of indel events (“gaps”) similar to that in the original data, we generated pre-aligned datasets by simulating them with the Lavalette Distribution model. We set the shape parameter α to 2.4 with an indel-rate of 0.01747 and imposed a maximum indel length of 400 site positions (see Section 3.1 in Supplementary File S1).

## Results

### Myriapod clades without Edafopoda

#### Unfiltered species-quartet support

**Figure 5.**
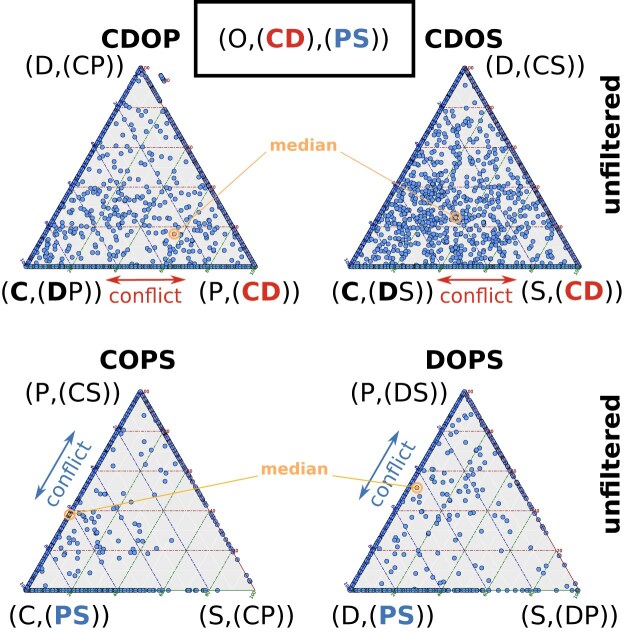
Unfiltered support values for species-quartet trees. Ternary plots with unfiltered support values for species-quartet trees (blue spots) that include the outgroup (O); their medians represent the average for the corresponding quartet of clades. O = outgroup, C = Chilopoda, D = Diplopoda, P = Pauropoda, and S = Symphyla. The Szucsich tree is (O,(CD),(PS)). The proximity of points to corners indicates the signal strength of each quartet in relation to the three possible quartet topologies. Median support values placed on edges between corners indicate that there is no distinct phylogenetic signal in the data. The analysis of quartets for CDOP and CDOS, assessing support for the sister-group relationship of Chilopoda and Diplopoda (“CD,” right triangle corner), demonstrates that there is no distinct signal and a substantial conflict between this relationship and the position of Diplopoda closer to the other taxa. Similarly, in the analysis of the COPS and DOPS quartet clade combinations, evaluating support for monophyletic Edafopoda “(PS),” there is no distinct preference for a sister-group relationship. The median support is slightly closer to the “CD” corner in CDOP and to “PS” in the COPS triangle.

The quartet analysis at clade level including the outgroup to assess support for the sister-group relationship of Chilopoda and Diplopoda “(CD group)” highlights a notable conflict between this relationship and the non-monophyly of Chilopoda and Diplopoda. In the unfiltered analyses Chilopoda appears either in moderate (CDOP) or strong conflict (CDOS) to be either closest to the outgroup or to Diplopoda. A similar conflict is observed for monophyletic Edafopoda “(Pauropoda + Symphyla)” in the analysis of the quartets COPS and DOPS, where Pauropoda is closer to the outgroup (Fig. 5). The median quartet support consistently revealed low values for each clade combination, ranging from zero (indicating no support) to 1 (representing full support). Notably, the highest support was observed for a topology featuring a monophyletic group of Chilopoda and Diplopoda, achieving 0.57 in CDOP, while the second-best support was recorded at 0.34 in CDOS. Edafopoda reached a top quartet support of 0.62 in COPS and 0.45 in DOPS, with the second-highest support observed for Edafopoda in DOPS (Table 3).

**Table 3.** Median support of quartet topologies using the clades Chilopoda (C), Diplopoda (D), Pauropoda (P), and Symphyla (S), as well as the group of outgroup species (O)^1^

Clade-Quartet	Tree	Unfiltered	“RISK”	“RISK+DIST”
CDOP	P,(CD)	⋆ 0.57	0.00	0.00
	**C,(D**P)	0.27	**0.78**	**0.79**
	D,(CP)	0.16	0.22	0.21
CDOS	S,(CD)	0.34	0.00	0.00
	**C,(D**S)	**0.41**	**0.82**	**0.84**
	D,(CS)	0.25	0.18	0.16
COPS	C,(PS)	• 0.62	• 0.91	• 0.92
	P,(CS)	0.38	0.09	0.08
	S,(CP)	0.00	0.00	0.00
DOPS	D,(PS)	0.45	• 0.87	• 0.88
	P,(DS)	⋄ 0.52	0.11	0.11
	S,(DP)	0.03	0.02	0.01

^1^We compare unfiltered quartet support values with support after application of the “RISK” and “DIST” filters (see “Materials and methods”), which delete topologies with the best support when they do not have a distinct signal in comparison with the alternatives for the same quartet. Predominating support for the sister-group relationship of Chilopoda and Diplopoda is only obtained in the unfiltered CDOP analysis (indicated by “⋆”). Conversely, in all analyses of filtered CDOP and CDOS topologies, as well as in the unfiltered CDOS analysis, CD monophyly is not supported, with Chilopoda placed next to the outgroup (highlighted in bold). Monophyletic Edafopoda “(PS)” become best supported across all quartet analyses (marked by “•”), except in the unfiltered analysis of DOPS, where a relationship “P,(DS)” is slightly stronger supported (marked by “⋄”). Significantly, the optimal quartet support increases substantially for both Chilopoda positioned closest to the outgroup, and for monophyletic Edafopoda when the “RISK” filter is implemented. This support becomes even slightly stronger when combined with the “DIST” filter approach.

**Figure 6.**
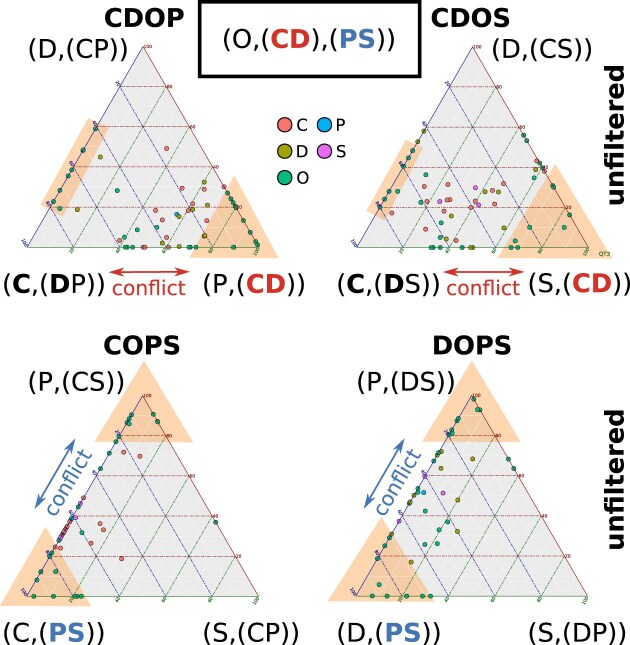
Ternary plots with quartet support at the level of species (abbreviations as in Fig. 5). The most notable contradictions (areas shaded in yellow) occur in the outgroup species (green dots), where some trigger strong support for a monophyletic group Chilopoda + Diplopoda (“CD”) in CDOP and CDOS, while others favor arrangements incompatible with a “CD” group. A similar pattern is observed for the monophyly of Edafopoda (“PS”) in COPS and DOPS. The majority of ingroup species produce no distinct support for a triangle corner, which indicates weak phylogenetic signal in the data set.

Analyzing the median support contribution of species in individual quartets within each clade-quartet reveals that conflicting signals are mainly influenced by specific outgroup taxa. The support for the majority of ingroup species-quartets in each clade-quartet analysis falls within the range of conflicting outgroup-supported tree scenarios (Fig. 6).

**Figure 7.**
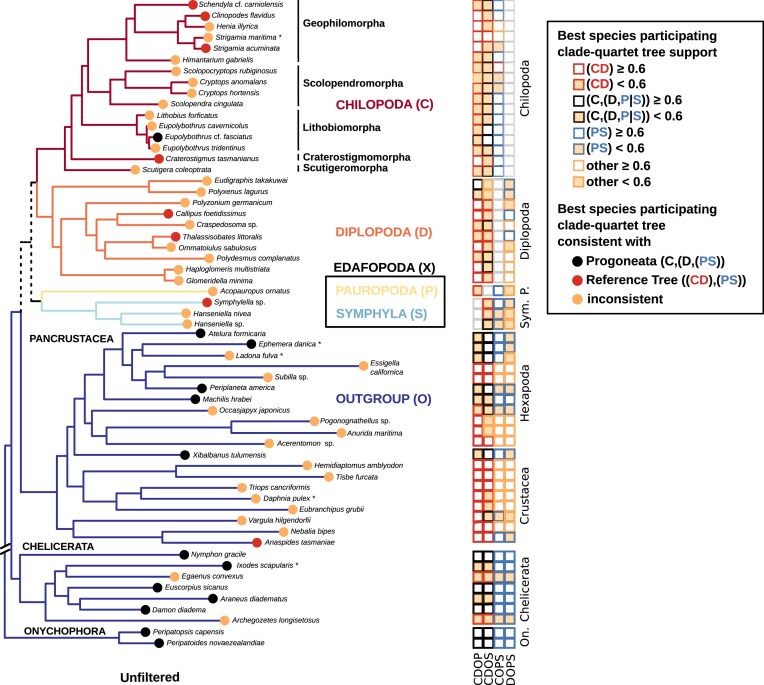
Overview of unfiltered clade-quartet support mapped on the reference tree of Szucsich et al. [3]. Black dots: quartets involving this species provide consistent clade-quartet support for the Progoneata hypothesis; red dots: If considering these species, the quartets support Chilopoda as siter-group to Diplopoda and monophyletic Edafopoda. Yellow dots: species-related quartet support across different clade-quartets is tree inconsistent. The rows of squares on the right side indicate for each species which best tree support the different clade-quartets get when this species is included. The squares refer to those clade combinations that include the outgroup (CDOP, CDOS, COPS, DOPS). Moderate support (less than 0.6) is shaded with yellow. Red squares represent the best clade-tree support consistent with the reference tree, black squares denote support for Chilopoda as the basal lineage in a rooted tree, blue squares indicate support for monophyletic Edafopoda, and yellow squares denote inconsistent support for the Reference Tree or for Progoneata.

When assessing the tree consistency of outgroup species-related support contributions in clade-quartets, only one crustacean outgroup species (*A. tasmaniae*) supported a sister-group relationship between Chilopoda and Diplopoda next to Edafopoda in all clade-quartet combinations. Generally, outgroup species with shorter root-to-tip branch lengths in the tree of Szucsich et al. [3] provide more consistently best quartet support contributions for Chilopoda as the earliest ingroup lineage and for the monophyly of Edafopoda. Moreover, as the root-to-tip branch distance of outgroup species increases, the occurrence of species with inconsistent overall tree support contributions for any larger clade-tree becomes more frequent across the best clade-quartet trees. The majority of crustaceans and hexapods with a longer root-to-tip branch distance therby tend to support a sister-group relationship between Chilopoda and Diplopoda, with less support observed for monophyletic Edafopoda. In contrast, the crustacean outgroup species with the shortest root-to-tip branch distance (*X. tulumensis*) consistently supports Chilopoda as the earliest ingroup lineage and the monophyly of Edafopoda. Both onychophorans and the majority of chelicerates also consistently exhibit the best clade-quartet support for this tree (Fig. 7).

The overall consistency of ingroup species-related best tree support contributions in clade-quartets does not correlate with different root-to-tip branch lengths of terminal taxa. Most ingroup species generally favor best trees in quartets of single clade combinations that do not conform with any tree encompassing all clades. In Chilopoda, the majority of Lithobiomorpha species support mutually incongruent clade topologies across different clade-quartets, as do the five species of Scolopendromorpha, the sole representative of Scuttigeromorpha (*S. coleoptrata*), and three out of six Geophilomorpha. In Diplopoda, most species also tend to support incongruent best trees in quartets of single clade combinations, a result shared by the solitary pauropod (*A. ornatus*) and two symphyla species. However, the quartet participations of one chilopod (*E. fasciatus*) and two diplopods in species-individual quartets result in overlapping support for Chilopoda as the earliest ingroup lineage and the monophyly of Edafopoda. In contrast, quartet participations of seven other ingroup species (four Chilopoda, two Diplopoda, and one Symphyla) consistently exhibit the best tree support contribution for a sister-group of Diplopoda and Chilopoda, adjacent to Edafopoda, in all four distinct clade-quartet analyses (Fig. 7).

#### Filtered clade-quartet support

**Figure 8.**
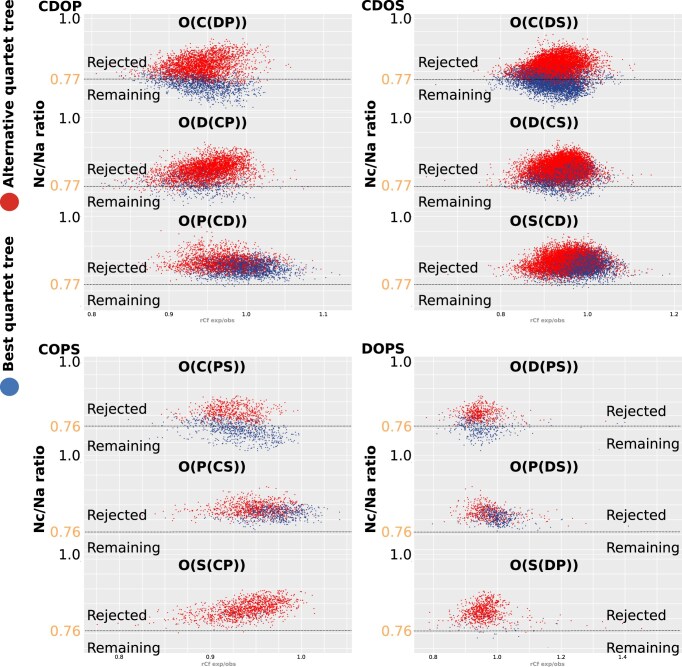
Distribution of convergent-to-apomorph tree signal in species-quartets. Graph showing support values for quartet topologies for the clade combinations (CDOP: top left; CDOS: top right; COPS: bottom left; DOPS: bottom right) with related support expressed as ratios of potentially convergent (“Nc”) to apomorphic (“Na”) character states (“Nc/Na ratio”; y-axis). The examined topologies are shown at the top of each clade combination related graph segment. The visual representation comprises clouds of dots, with each dot symbolizing the Nc to Na proportion for a distinct species-quartet-related clade topology. Blue dots signify that this topology is the best-supported in a species-quartet, while red dots represent species-quartets where this topology is supported as alternative, second or third best tree. Within each clade combination, a dashed horizontal line indicates the optimized upper threshold limit for Nc to Na ratios set by the “RISK” filter (yellow values; y-axis). This line acts as a delineation between ratios of Nc to Na character states for each species-quartet’s best tree that are considered to be more reliable (below the line) and values that are refused (above the line). The aim is to keep a balance between rejected and remaining quartets in each clade combination by preserving as many best trees (blue dots) of high IC, expressed by a low Nc to Na proportion, as possible without loosing too many quartets across a clade combination. For this purpose, we use a common function, which is determined through an adapted uphill climbing algorithm, as detailed in the study by Misof et al. [60] (see Section 1.4.3 and Section 2 in Supplementary File S1). After the “RISK” filtering, the best trees of the retained species-quartets in a clade combination exhibit an Nc to Na proportion less likely for this clade-tree if an alternative tree is best supported in species-quartets. This places them in a range with reduced noise impact, visually depicted by clouds of blue dots below the threshold line, which are less mixed with red dots. The best trees of rejected species-quartets do not meet this criterion. Analyzing the Nc/Na ratio of the retained quartets indicates that most best trees favor Chilopoda as the earliest lineage within the ingroup for CDOS (“O(C(DP))”) and CDOP (“O(C(DS))”), while the number of best trees supporting a sister relationship of Chilopoda and Diplopoda (“O(P(CD))” and “O(S(CD))”) is consistently low. In the case of COPS (bottom left) and DOPS (bottom right), the majority of best trees identified for the retained quartets distinctly support the monophyly of Edafopoda (“O(C(PS))” and “O(D(PS))”). Simultaneously, the best trees representing alternative configurations, particularly those with a weak signal, are predominantly rejected in both clade combinations (middle and bottom of each clade combination related graph segment).

For every clade combination, the carefully optimized upper threshold of the “RISK” filter effectively determined the ratios of potentially misleading convergent (“Nc”) to apomorphic (“Na”) character states in species-quartets. The lower this ratio, the higher the proportion of putative apomorphies, representing the split-supporting phylogenetic signal (see Section 1.4 and analysis corresponding results of Section 2 in Supplementary File S1). Quartets that fall below this threshold for the best tree are considered appropriate for the final tree assembly, resulting in the retention of quartets with best tree’s supported in a range of putative apomorphies, which has been identified to be less prominent for weak trees (filter results for the clade-quartets CDOP, CDOS, COPS, DOPS are shown in Fig. 8).

**Figure 9.**
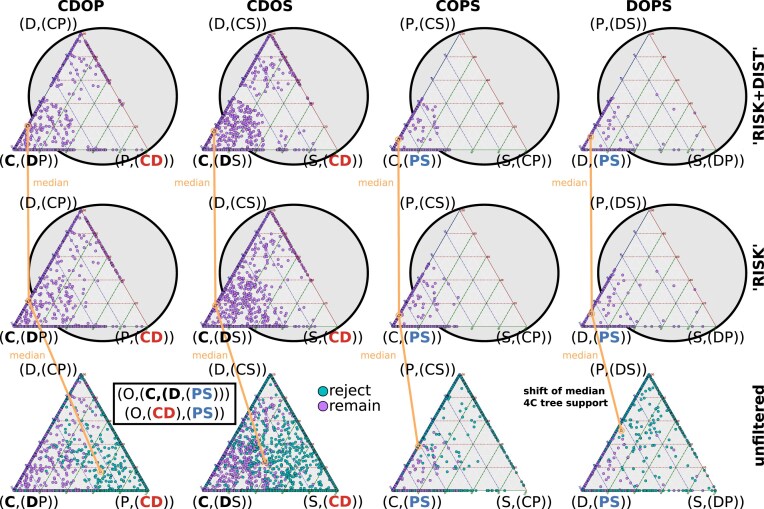
Filtered versus unfiltered species-quartet tree support. Ternary plots, as depicted in Fig. 5, show how species-quartet topologies support quartet topologies for each clade combination that includes the outgroup (indicated in triangle corners). The application of the “RISK” filter individually and in combination with the “DIST” filter (“RISK+DIST”), is compared with unfiltered topologies (horizontal rows of triangles). In contrast to the unfiltered analyses, the clouds of dots are less dispersed in the filtered analyses, being attracted to the left corner where the clade Chilopoda (C) is predominately supported as the earliest ingroup lineage (CDOP and CDOS) or where Pauropoa (P) and Syphyla (S) are sister-groups (“PS” in COPS and DOPS). The addition of the “DIST” filter (upper row of triangles) has a less pronounced impact on the median support.

Thus, filtering quartets based on the Nc/Na ratio significantly reduces the occurrence of best tree conflicts in each of the four clade combinations. The majority of “RISK” filtered quartets support Chilopoda as the first lineage in CDOP and CDOS and monophyletic Edafopoda in DOPS and COPS. In contrast, quartets supporting Chilopoda as sister-group to Diplopoda “(CD)” are strongly reduced in the analysis (Fig. 9).

**Figure 10.**
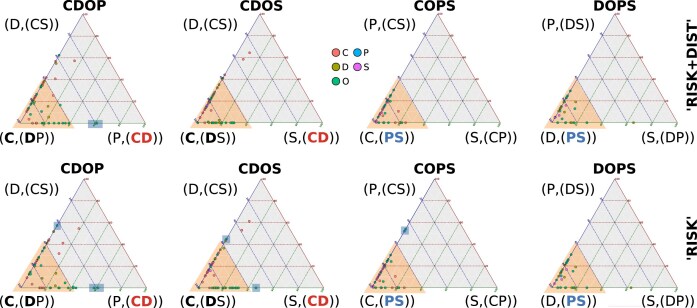
Contribution of species to filtered clade-quartet tree support. Ternary plots showing median quartet tree support for species individual quartet participations after filtering with “RISK” or “RISK+DIST” (rows of triangles). Emphasizing the instances highlighted in yellow, excluding specific outgroup species (highlighted by blue squares), along with certain chilopods and diplopods (dots not highlighted in green or yellow) in CDOS and CDOP clade combinations, the majority of species in each clade combination provide robust support. The support predominantly favors either Chilopoda as the first lineage (“C(DP)” and “C(DS)”) or monophyletic Edafopoda (“PS”), with minimal preference for alternative topologies. The inclusion of the “DIST” filter marginally enhances the signal for this relationship, particularly affecting the support of outgroup species.

Analyzing the support distribution of retaining quartets for individual species, in the “RISK” filtered analyses the quartet-tree support transitioned from a conflicted tree support to a robust signal for Chilopoda as the basal lineage of the ingroup in CDOS and CDOP. There was an improvement in signal strength contribution for both ingroup and outgroup species, predominantly coinciding with the same tree topology. Similarly, monophyletic Edafopoda are supported in COPS and DOPS clade combinations (Fig. 10).

**Figure 11.**
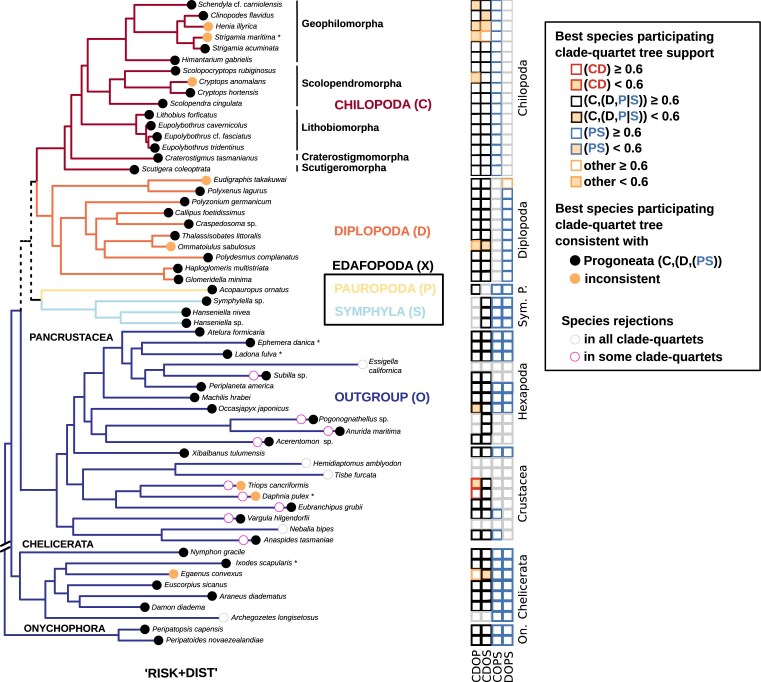
Fit of species-related best tree support in individual quartets for clade-quartet topologies mapped onto the tree of Szucsich et al. (2020). Black dots represent species with individual best four-clade tree support contributions compatible with the Progoneata hypothesis, while yellow dots represent species with individual best four-clade tree support contributions incompatible with any tree encompassing all clades. Species with individual best four-clade tree support contributions compatible with a monophyletic group of Chilopoda + Diplopoda are absent after applying the quality filtering with “RISK” and “DIST” filters. Purple-circled squares indicate that quartets with this species are entirely rejected in some clade combinations, while gray-circled species are rejected in all clade analyses. Squares on the right side summarize the fit of species to quartets of rooted clades (CDOP, CDOS, COPS, DOPS, as in Fig. 6). Each species’ best quartet-topology support is denoted by color-coded squares, reflecting support quality and clade pairing. Red squares: best quartet-tree support consistent with monophyletic Chilopoda + Diplopoda (“(CD)”), black squares: support for Chilopoda as the first lineage (“C(DP)” or “C(DS)”); blue squares: support for monophyletic Edafopoda (“PS”); yellow squares: inconsistent four-clade tree support. Low or moderate support contribution of species (less than 0.6) is additionally shaded in yellow. Species with no remaining quartet participations in clade combinations after the quartet filtering are coded by gray scales (see caption). The analyses are dominated by species quartets consistently supporting Progoneata, with Chilopoda as the basal lineage (support greater/equal than 0.6) and with monophyletic Edafopoda (represented by black dots along the tree), outnumbering those with inconsistent support (yellow dots). Full consistency with a Chilopoda + Diplopoda clade is not observed.

Analyzing quartet rejections for individual species’ participation reveals that a majority of quartets are discarded by the “RISK+DIST” filter when outgroup species with longer root-to-tip branch lengths are involved. This is particularly pronounced in hexapods and crustaceans, where the total number of quartets remaining after filtering diminishes for many of these species to zero, leading to complete rejections in single or all clade combinations that include specific outgroup species. In contrast, most quartets with less derived “short-branched” species are retained (Table 4). As a result, four out of six remaining crustaceans, five out of six remaining chelicerates, and all remaining hexapods and onychophorans consistently show best support for topologies with the Progoneata group. Contrary, only two closely related crustacean species (*T. cancriformes* and *D. pulex*) still trigger the sister-group relationship of Chilopoda and Diplopoda in the CDOP configuration, while all other quartet analyses do not support this relationship (Fig. 11).

**Table 4.** Percentage of quartet participations remaining for each outgroup species in each clade-quartet analysis after “RISK” filtering. Generally, the more derived a species is (the longer the branches leading to it), the lower is its percentage of retained quartet participations. For the two “short-branched” Onychophora, as well as the three less derived Chelicerata species, the majority of their quartets is retained (75% to 100%). In contrast, the quartet participation for the two most derived chelicerates, *A. longisetosus* and *I. scapularis*, is strongly or completely reduced. Except for the less derived *X. tulumensis*, the closest crustacean relative of the hexapods in the tree of Szucsich et al. [3], quartet participations of crustacean species are strongly reduced (≤ 10% of quartets retained) or completely rejected. The same occurred with the most derived hexapod species. Species without any quartet participation left are marked by a “⋆”

Clade	OTU	Subgroup	CDOP	CDOS	COPS	DOPS
Out	*Araneus_diadematus*	Chelicerata	49.38	27.71	56.25	23.33
Out	*Archegozetes_longisetosus* ⋆	Chelicerata	0.00	0.21	0.00	0.00
Out	*Damon_diadema*	Chelicerata	99.38	94.79	100.00	96.67
Out	*Egaenus_convexus*	Chelicerata	90.62	69.79	91.67	70.00
Out	*Euscorpius_sicanus*	Chelicerata	94.38	75.83	97.92	86.67
Out	*Ixodes_scapularis*	Chelicerata	14.37	5.42	4.17	16.67
Out	*Nymphon_gracile*	Chelicerata	36.25	33.33	68.75	43.33
Out	*Anaspides_tasmaniae* ⋆	Crustacea	10.00	4.58	8.33	0.00
Out	*Daphnia_pulex* ⋆	Crustacea	1.88	2.71	0.00	0.00
Out	*Eubranchipus_grubii* ⋆	Crustacea	1.25	1.88	6.25	0.00
Out	*Hemidiaptomus_amblyodon* ⋆	Crustacea	0.00	0.00	0.00	0.00
Out	*Nebalia_bipes* ⋆	Crustacea	0.00	0.00	0.00	0.00
Out	*Tisbe_furcata* ⋆	Crustacea	0.00	0.00	0.00	0.00
Out	*Triops_cancriformis* ⋆	Crustacea	7.50	5.83	2.08	0.00
Out	*Vargula_hilgendorfii* ⋆	Crustacea	8.12	7.08	8.33	0.00
Out	*Xibalbanus_tulumensis*	Crustacea	51.25	47.92	68.75	50.00
Out	*Acerentomon_maius* ⋆	Hexapoda	7.50	5.62	0.00	0.00
Out	*Anurida_maritima* ⋆	Hexapoda	0.00	0.21	0.00	0.00
Out	*Atelura_formicaria*	Hexapoda	65.62	52.71	72.92	36.67
Out	*EDANI*	Hexapoda	28.12	18.12	29.17	6.67
Out	*Essigella_californica* ⋆	Hexapoda	0.00	0.00	0.00	0.00
Out	*LFUL*	Hexapoda	43.12	43.33	66.67	16.67
Out	*Machilis_hrabei*	Hexapoda	54.37	47.08	54.17	63.33
Out	*Occasjapyx_japonicus_neu*	Hexapoda	31.87	48.12	50.00	16.67
Out	*Periplaneta_americana*	Hexapoda	63.75	52.08	72.92	43.33
Out	*Pogonognathellus_sp* ⋆	Hexapoda	0.00	0.21	0.00	0.00
Out	*Subilla_sp* ⋆	Hexapoda	9.38	4.58	0.00	0.00
Out	*Peripatoides_novaezealandiae*	Onychophora	75.62	63.33	97.92	63.33
Out	*Peripatopsis_capensis_F2014*	Onychophora	90.62	75.00	100.00	90.00

Compared to the outgroup, ingroup species participations are less strongly reduced in clade-quartets, with remaining quartet participations ranging between approximately 16% and approximately 50%, congruent with the root-to-tip branch length of each species (Table 5). Inconclusive topology preferences persist when three Chilopoda (two Geophilomorpha, one Scolopendromorpha) and two Diplopoda (ingroup species) are involved in species-quartets (Fig. 11).

**Table 5.** The percentage of quartet participations retained for each ingroup species in each clade-quartet analysis after “RISK” filtering varies between approximately 16% and approximately 50%, depending on the degree of derivation (root-to-tip distance in tree) of the species. Generally, within each ingroup clade, species participations tend to be less extremely reduced compared to the outgroup species (Table 4)

Clade	OTU	CDOP	CDOS	COPS	DOPS
Chil	*Clinopodes_flavidus*	20.69	15.29	21.84	—
Chil	*Craterostigmus_tasmanianus*	32.07	26.55	40.23	—
Chil	*Cryptops_anomalans*	31.72	19.31	35.63	—
Chil	*Cryptops_hortensis_F2014*	29.31	21.03	31.03	—
Chil	*Eupolybothrus_cavernicolus_sp*	45.86	41.38	50.57	—
Chil	*Eupolybothrus_cf_fasciatus*	52.07	52.07	50.57	—
Chil	*Eupolybothrus_tridentinus*	46.90	41.61	50.57	—
Chil	*Henia_illyrica*	18.62	15.29	16.09	—
Chil	*Himantarium_gabrielis*	27.59	20.23	40.23	—
Chil	*Lithobius_forficatus*	42.41	38.97	47.13	—
Chil	*Schendyla_cf_carniolensis*	21.03	16.09	21.84	—
Chil	*Scolopendra_cingulata*	35.17	30.80	45.98	—
Chil	*Scolopocryptops_rubiginosus*	25.86	27.13	43.68	—
Chil	*Scutigera_coleoptrata*	43.45	43.22	52.87	—
Chil	*Strigamia_acuminata*	20.00	14.25	18.39	—
Chil	*Strigamia_maritima*	22.76	11.26	16.09	—
Dipl	*Callipus_foetidissimus*	25.22	24.64	—	41.38
Dipl	*Craspedosoma_sp*	28.02	26.72	—	33.33
Dipl	*Eudigraphis_takakuwai*	55.17	39.22	—	16.09
Dipl	*Glomeridella_minima*	23.28	26.72	—	19.54
Dipl	*Haploglomeris_multistriata*	26.29	27.95	—	21.84
Dipl	*Ommatoiulus_sabulosus*	31.47	24.64	—	36.78
Dipl	*Polydesmus_complanatus*	40.73	37.72	—	17.24
Dipl	*Polyxenus_lagurus*	42.89	32.97	—	11.49
Dipl	*Polyzonium_germanicum*	26.29	18.10	—	20.69
Dipl	*Thalassisobates_littoralis*	22.84	12.86	—	31.03
Paur	*Acopauropus_ornatus*	32.22	—	36.42	24.94
Symp	*Hanseniella_nivea*	—	21.70	35.13	20.34
Symp	*Hanseniella_sp*	—	32.56	35.99	23.10
Symp	*Symphylella_sp*	—	27.20	38.15	31.38

While the “DIST” filter once again reduces the number of retained quartets, its additional impact is less pronounced. However, it slightly altered the overall signal strength in quartets with ougroup species, by shifting the support of species-quartets with the more derived crustacean *E. grubii* towards Progoneata. Additionally, it resulted in the complete rejection of species-quartets of the strongly derived chelicerate A. longisetus (Figs 9 and 10).

#### Rooted quartet support in final clade-tree analyses

The unfiltered analyses resulted in a best rooted tree consistent with the results of Szucsich et al. [3]. However, unfiltered clade-quartet analyses expose notable conflicts with alternative topologies, as evidenced by the disparity in final support between the best rooted clade-tree and the next two best supported trees. The final support of the best rooted clade-tree resulted from unfiltered data, is signifcantly exceeded by the final support favouring Progoneata with monophyletic Edafopoda (“O(C(D(PS)))”) in the filtered analyses, with a slight improvement in both best tree support and support distance to the next two best topologies if the “RISK” and “DIST” filters are used combined (Table 6).

**Table 6.** The final support for the top three rooted clade-trees, derived from unfiltered and filtered species-quartet topologies (filters “RISK,” “RISK+DIST”). In the unfiltered data, the best tree supports the clade Chilopoda + Diplopoda and Edafopoda (labeled by “⋆”), but the support differences to the next best trees (Δ_Best|2nd_, Δ_Best|3rd_) are relatively small, indicating a notable conflict among these three topologies. However, in the filtered analyses, the support for the best tree, favoring Progoneata and monophyletic Edafopoda, is significantly stronger (highlighted in bold), and the support difference from the alternative topologies is larger

Final Support	Best	2nd Best	3rd Best	Δ_Best|2nd_	Δ_Best|3rd_
Unfiltered	⋆ 5.91	5.27	5.16	0.64	0.75
“RISK”	**8.66**	7.05	6.83	1.61	1.83
“RISK+DIST”	**8.78**	7.15	6.91	1.63	1.87

### Myriapod clades with Edafopoda

**Figure 12.**
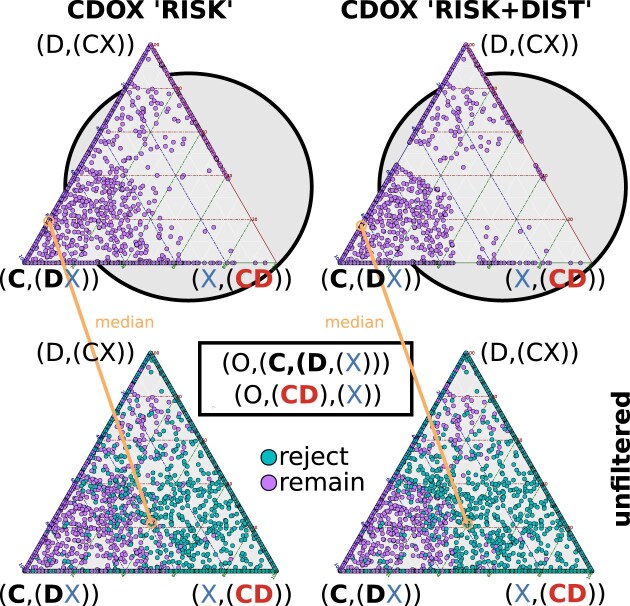
Species-quartet support with Edafopoda. Ternary plots with support values for species-quartets and their medians (highlighted in yellow), both without filtering (bottom) and with filtering (top: utilizing “RISK” and the combined “RISK+DIST” filters), now considering the clade Edafopoda (clade X). In comparison to the unfiltered analysis, the “RISK” filtered analyses of CDOX (bottom) exhibit a substantial loss of signal strength for the group Chilopoda + Diplopoda “CD” (right triangle corner). While the “DIST” filter reduces species quartets with weak signal strength, its additional impact on the median support is less pronounced. It is crucial to note that this test cannot confirm the monophyly of Edafopoda.

Combining Pauropoda and Symphyla into the clade Edafopoda (X), the outcomes of this clade-quartet analysis (CDOX) closely mirrored those previously described for the rooted clade topologies. Unfiltered quartet analyses revealed substantial conflict between a Chilopoda + Diplopoda clade and Chilopoda as the earliest lineage. Quartet filtering notably strengthened support for the latter relationship while diminishing support for Chilopoda + Diplopoda (Fig. 12). Calculating the median species-quartet support once again showed conflicts in the unfiltered analysis, primarily arising from varying strong support in different combinations with outgroup species. However, robust support for Progoneata emerged when the data underwent quartet filtering (see Section 2.4 in Supplementary File S1).

The improvement in signal quality favoring Progoneata in filtered quartets is also evident in the score difference among the three quartet trees (see Section 2.4.6 in Supplementary File S1). The support for the unfiltered best topology, favoring the clade Chilopoda + Diplopoda, is nearly identical to the support for Progoneata in the second-best tree. However, after rejecting low-quality quartets, Progoneata is twice as strongly supported in the best tree compared to the best tree of the unfiltered data, while the clade Chilopoda + Diplopoda receives zero support among the third-best topologies.

### Simulations

**Figure 13.**
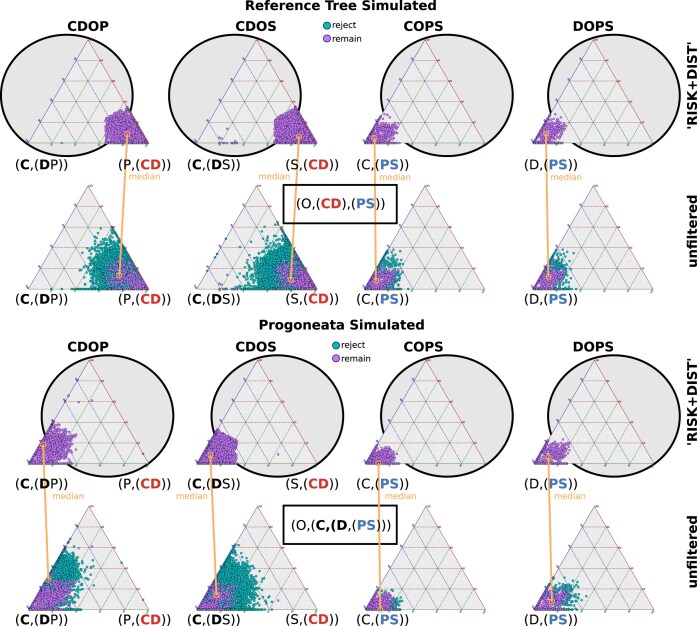
Species-quartet support in sequence simulations. Ternary plots showing examples of species-quartet support in sequence data with indels simulated on the reference tree (Szucsich et al., 2020) (upper two rows) and on a topology with monophyletic Progoneata and Edafopoda (lower two rows), both with and without the application of quartet filters “RISK+DIST.” In both trees, there is considerably less noise and conflict in the simulated data compared to analyses of the real dataset (compare with Fig. 9), making the filters less influential.

The simulations affirm the reliability of our method. Quartet analyses for the simulated data, whether based on the original tree of Szucsich et al. [3] or data derived from a topology with monophyletic Progoneata, with Chilopoda simulated as the first ingroup lineage, and monophyletic Edafopoda consistently demonstrate strong signal for the topology used to generate the sequences. This is consistent across both complete and incomplete (with Indels) character simulations, as well as scenarios with different numbers of defined clades (outgroup plus four clades with separated Pauropoda and Symphyla or three clades with monophyletic Edafopoda). The signal strength tends to be slightly increased when quartets have been filtered (see Section 3 in Supplementary File S1 for final four clade support). Example ternary plots for analyzed quartet support before and after the filtering process are shown in Fig. 13 and Supplementary File S1.

## Discussion

Our analysis sought to clarify the evolutionary relationships among the four major myriapod subgroups (Chilopoda, Diplopoda, Pauropoda, Symphyla) using the second-codon-only dataset recently published by Szucsich et al. [3]. The two competing hypotheses under consideration were the sister-group relationship of Chilopoda with Diplopoda [3] versus the monophyly of Progoneata (Diplopoda with Edafopoda).

Our methodological approach, incorporating the PhyQuart algorithm and subsequent steps of filtering and a quartet-based supertree approach (see Section 1 in Supplementary File S1), is quite different from conventional probabilistic and distance methods currently used in molecular phylogenetics. PhyQuart is based on the analysis of site patterns [45]. By focusing on polarized quartets of sequences that include outgroup taxa, our method allows for non-heuristic (exact) Hennigian analysis in larger trees and the evaluation of quartets drawn from sets of multiple-sequence clades.

We developed filters that reduce negative effects of chance similarities and plesiomorphic homologies. Major advantages are (i) that putative plesiomorphic character states are excluded as evidence for monophyly, (ii) the misleading effects of long branches are eliminated empirically sequence by sequence, (iii) alignment sites with missing data do not have to be deleted over the whole alignment but are excluded only at the level of sequence quartets, and iv) the user may obtain detailed information on the quality of phylogenetic signal contributed by single sequences and species. A disadvantage is that the analysis of site patterns occurs at the level of sequence quartets. There is no comparable method for the analysis of site patterns across more than four taxa.

In the study presented here, unfiltered quartet analyses indicate support for the monophyly of the group Chilopoda + Diplopoda, suggesting a shared evolutionary history between these subgroups as suggested by Szucsich et al. [3]. This challenges the traditional perspective that often positions Chilopoda as a distinct early branch in myriapod evolution, opposed to the taxon Progoneata, as proposed by Fernandez et al. [2] with monophyletic Dignatha or by Zwickl et al. [5] with monophyletic Edafopoda (Pauropoda and Symphyla). The presence of tree conflict in our unfiltered analyses, particularly for quartets with various strongly derived outgroup species, and the relatively small support difference between the best clade-tree to the next best one also raise questions about myriapod subgroup relationships and about data quality.

Our unfiltered analyses of myriapod subgroup relationships enable a comprehensive exploration of the varying tree signal quality in quartet data. Thereby the lack of support for monophyletic Dignatha (Pauropoda and Diplopoda; Fernandez et al. [2]) and the consistent presence of partial support for both monophyletic Edafopoda and the Chilopoda + Diplopoda group raise more questions about the position of the root. Considering the consistently low overall signal for monophyletic Dignatha (Pauropoda + Diplopoda) in our analyses, this classification appears unlikely. This observation aligns with the findings reported by Szucsich et al. [3]. However, the best unfiltered tree is susceptible to noise and conflicting signals and therefore unreliable.

The incorporation of filters for weak species-quartets improves the impact of more distinct tree signals in the data. The “RISK” filter (see Supplementary File S1) assesses the ratio between potential apomorphies and convergent (non-homologous) signals, providing a nuanced selection of site pattern information in sequence quartets. It effectively resolves tree conflicts by rejecting species-quartets within ranges where the ratios of apomorphic to convergent signals are similar for the same clade combination. As shown by our results, this strategy can be particularly effective in resolving conflicts between quartet topologies and between the tree support contributed by different species. Simultaneously, the “DIST” filter directly measures conflict between alternative quartet trees, providing a quantitative measure for the number of conflicting characters. While the “DIST” filter effectively resolves conflict among topologies of clade-quartets with relatively low support, its impact on the final best clade-tree support is modest. Nevertheless, the combined use of both “RISK” and “DIST” filters enhances the analysis, offering improved discrimination between signal qualities in single quartets and strengthening the reliability of selected trees.

After rejecting species-quartets with low support or strong conflict, our filtered analyses consistently favored Chilopoda and Progoneata. This “Chilopoda first” scenario is obtained with or without monophyletic Edafopoda in the sister-group. Filtering led to a substantial reduction of conflicting signal-like patterns contributed mainly by derived outgroup species, resulting in significantly stronger support for the relationship proposed by Zwickl et al. [5]. Importantly, there is also a notable difference in support for the final rooted-clade tree compared to the next best tree.

In our case study, we also get an enhanced signal for monophyletic Edafopoda. This challenges the assertion made by Fernandez and colleagues that support for a clade Edafopoda (Pauropoda + Symphyla) found in other studies could be attributed to artifacts, particularly LBA of Pauropoda towards the equally long-branched Pancrustacea, including crustaceans and hexapods [3].

Our findings provide insights into the potential impact of long-branch artifacts (LBA) on phylogenetic reconstructions. The susceptibility of unfiltered analyses to conflicting signals and noise, as demonstrated in our study, highlights the challenges posed by long-branch taxa.

Interestingly, partial or complete filtering of weakly supported sequence quartets automatically affects outgroup species with longer root-to-tip branches (comprising in the present study almost all crustaceans, many hexapods, and one chelicerate). Long branches are problematic, because a clade’s apomorphic character states inherited from a common ancestor can be substituted, which implies a loss of relevant phylogenetic information. Also, substitutions along branches of outgroup species can superimpose characters that are needed to identify older homologies that are plesiomorphic in ingroup taxa. The effect can be that some ingroup taxa share older similarities that are no evidence for monophyly but which attract the affected branches. The reason why in our analyses many outgroup species prove to be problematic is their older radiation age, in addition to differences in substitution rates. The radiation of ingroup taxa that are derived from a last common ancestor is always younger than the root-to-tip age of outgroup species.

Empirical datasets often violate SRH conditions due to their inherent complexity and heterogeneity. While these violations may raise concerns, we believe that SeaLion’s design is well-suited to handle such data. By aggregating support across multiple quartets, SeaLion minimizes the impact of SRH violations and can still extract meaningful phylogenetic signals from complex datasets. The strong performance observed in our simulations supports SeaLion’s robustness under evolutionary scenarios, suggesting its reliability even when data deviate from idealized SRH assumptions. While SeaLion focuses on minimizing the effects of conflicting signals by aggregating support scores across multiple quartets, it achieves this granularity by identifying “good sequences” rather than “good genes.” This sensitivity enables SeaLion to extract strong phylogenetic signals from subsets of sequences, even within genes where the majority of sequences exhibit low phylogenetic signal. This capability allows SeaLion to retain and leverage valuable information that might otherwise be excluded in methods that rely solely on “good genes.”

However, dealing with compositional heterogeneity among sequences remains a challenge for SeaLion. Methods such as the frequency-dependent significance test introduced by Steel et al. [61] and later generalized for maximum parsimony [62] may offer complementary insights for addressing these challenges. Future adaptations of these approaches could potentially enhance SeaLion’s ability to mitigate the effects of compositional biases, further improving its utility for analyzing complex and heterogeneous datasets.

Another feature of our method is its efficiency in evaluating and reconstructing internal branches between predefined clades of multiple species. This enables in our example a more targeted analysis of specific relationships within Myriapoda, shedding light on intricate details that might be overlooked in broader analyses. A disadvantage is that phylogenies within these selected clades require a new analysis, for which other, smaller well corroborated clades (e.g. at the level of families) have to be selected. The results can be subsequently combined to a supertree. However, when the selected clades in reality are not monophyletic, the analysis will be erroneous.

Simulated data further supported the effectiveness of our approach. Regardless of whether the simulations were character complete or incomplete (with Indels), our method consistently demonstrated strong signal for each underlying tree. The use of quartet filters enhanced the analyses by reducing conflicting signals and potential noise. The overall less noisy results from our simulations compared to the empirical data could be attributed to several factors, including potential alignment errors in the original data. Another contributing factor might be differences in gap distribution patterns. Although we made considerable efforts to match the gap distribution in our simulations to the empirical data by carefully tuning gap-related parameters, some discrepancies may still persist.

However, the success in maintaining robust results across both empirical and simulated datasets validates the reliability and versatility of our approach.

## Conclusion

To investigate the evolutionary relationships among major myriapod subgroups, our study utilized a unique divide-and-conquer quartet approach combining the PhyQuart algorithm with new quartet-based filters and supertree algorithms. Based on split-supporting site patterns and focusing on rooted quartets of sequences with polarized character states we get a non-heuristic Hennigian analysis of myriapod sequences. The result (“Chilopoda first” scenario) is compatible with morphology-based phylogenies. Our approach enables a comprehensive exploration of putative phylogenetic signal in larger trees using quartet topologies drawn from multiple-sequence clades. This innovative method enhances our ability to unravel complex evolutionary scenarios and contributes to a more precise understanding of phylogeny.

For Myriapoda, the rejection of a Diplopoda + Chilopoda group and the support for Progoneata with Edafopoda challenge previous assumptions. The comparison of unfiltered and filtered quartet topologies underscore the importance of methodological choices in phylogenetic analyses. The implementation of advanced filters for noise and misleading plesiomorphic characer states is pivotal for unraveling distinct signals and fortifying the robustness of phylogenetic reconstructions. This encompasses the acknowledgment and alleviation of the impacts of long branch artefacts in phylogenetic analyses. Further methodological research, considering additional datasets and refining the workflows, will continue to improve phylogenetic analyses.

## Supplementary Material

lqaf018_Supplemental_File

## Data Availability

SeaLion, a comprehensive manual, and a container file with all external script dependencies can be freely downloaded from: https://github.com/PatrickKueck/SeaLion.git and via Zenodo, DOI: 10.5281/zenodo.14865632.
