# Supplementary material for: Unraveling myriapod evolution: sealion, a novel quartet-based approach for evaluating phylogenetic uncertainty
Source: NAR Genom Bioinform. 2025 Mar 7;7(1):lqaf018. doi: 10.1093/nargab/lqaf018 (PMC11886814; doi:10.1093/nargab/lqaf018)
Supplement: lqaf018_Supplemental_File [file lqaf018_supplemental_file.pdf]

Patrick Kück, Mark Wilkinson, Juliane Romahn, Nathan I. Seidel, Karen Meusemann, Johann W. Wägele

Unraveling Myriapod Evolution - Supplementary File

January 27, 2025

Contents

1 SeaLion – Concept **1**
1.1 General Overview 1
1.2 PhyQuart-mapping 3
1.3 Supertree-approach (Icebreaker) 3
1.4 Quartet Filtering 5
1.4.1 'RISK' Filter 5
1.4.2 'DIST' Filter 6
1.4.3 Threshold Optimization 7

2 Myriapoda Subgroup Relationships **9**
2.1 SeaLion Parameter Specifications 9
2.2 Clade Assignments 10
2.3 Clade Analysis without Edafopoda 12
2.3.1 Filter Optimization 12
2.3.2 Clade-Quartet Related Tree Support 14
2.3.3 Species Related Quartet Participations 16
2.3.4 Species Related Clade-Quartet Tree Support Contribution 21
2.4 Clade Analysis with Edafopoda 24
2.4.1 Filter Optimization 24
2.4.2 RISK Filter Impact on Species-Quartets 25
2.4.3 Clade-Quartet Related Tree Support 27
2.4.4 Species Related Quartet Participations 27
2.4.5 Species Related Clade-Quartet Tree Support Contribution 30
2.4.6 Final Clade-Tree Support 33

3 Simulations **35**
3.1 Simulation Parameter 35
3.2 SeaLion Parameter Specifications 35
3.3 Final Clade-Tree Support without Edafopoda 36
3.4 Final Clade-Tree Support with Edafopoda 37

Bibliography **39**

CHAPTER 1

SeaLion – Concept

1.1 General Overview

Built upon the PhyQuart algorithm [1], SeaLion offers precise and non-heuristic quartet analyses involving multiple species-quartets. In SeaLion, individual outgroup-rooted tree support assessments for each clade-quartet are computed by averaging (using the median) results from numerous quartets of species. Users are required to define clades and specify an outgroup (for details on myriapod analyses, refer to section 2.2). Clades represent groups of species assumed to form confirmed monophyla (e.g., species of Chilopoda). It is important to note that phylogenies within these clades are not studied at this stage; a separate analysis would be necessary to explore subgroups within a given clade. SeaLion efficiently calculates collective scores for all possible clade-quartet arrangements rooted with an outgroup.

The SeaLion workflow (Figure 1.1) is structured into three hierarchical processes: (1) the analysis of individual species-quartets using the PhyQuart algorithm, (2) the amalgamation of single inferred PhyQuart scores from multiple species-quartets for a given clade-quartet through the PhyQuart-mapping algorithm (section 1.2), and (3) the inference of relationships among more than four predefined clades employing the supertree (Icebreaker) algorithm (section 1.3). While the original PhyQuart algorithm has been comprehensively described in a previous work [1], both the PhyQuart-mapping and Icebreaker algorithms represent novel developments that expand the potential utility of PhyQuart scores. These extensions aim to reveal and potentially resolve conflicting signals within the alignment.

SeaLion provides two distinct filtering methods to eliminate species-quartets of single clade-quartets with weak tree signal (see section 1.4). A single quartet filter, referred to as 'RISK', assesses for a quartet of species the distribution of PhyQuart scores for the three possible species-quartet corresponding clade-quartet topologies and excludes the quartet when the score exceeds a low-quality threshold (section 1.4.1). The second species-quartet filter, labeled 'DIST', measures the score distance between the top-ranked and the second-best quartet topology. It discards all quartets with a score difference falling below a specific threshold (section 1.4.2). Both filter thresholds are individually optimized using a tailored uphill-climbing algorithm integrated into SeaLion (section 1.4.3).

Due to the wealth of information provided at each analysis stage (all tables and figures in this supplement originate from the script itself; see the manual for a more detailed explanation of possible output files), SeaLion empowers us to meticulously assess the potential introduction of deceptive signals within the data that could impede accurate phylogenetic inferences. This thorough assessment affords us a more intricate grasp of the delicate balance between support and conflict within the examined hypotheses concerning the potential relationships among myriapod subgroups.

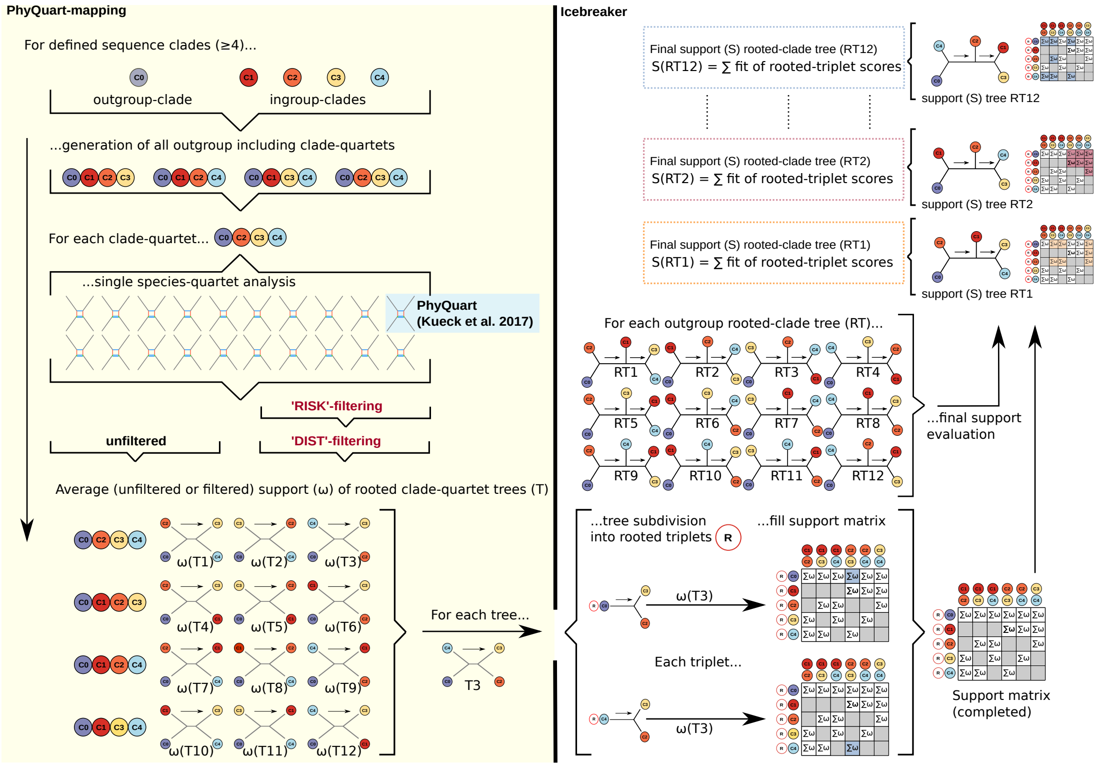

Figure 1.1: Overview of individual SeaLion processes. Left side: PhyQuart-mapping. For a predefined set of clades, each comprising four or more sequences, SeaLion systematically generates all conceivable outgroup-rooted clade-quartet combinations (top left). In the subsequent process (middle left), SeaLion analyzes species-quartets corresponding to clade-quartets, using the PhyQuart algorithm (shaded blue). Each species-quartet is characterized by a unique combination, involving one representative species from each clade, contributing to the clade-quartet. Within PhyQuart-mapping, the PhyQuart algorithm is then applied to derive comprehensive tree signal insights from these analyses. If specified, the 'RISK' and/or 'DIST' filter is employed on species-quartets. Regardless of the selected filter approach, the data is consistently analyzed separately in its unfiltered state. In the final Icebreaker process (right side), average support for each analyzed clade-quartet tree is transferred into a score matrix of rooted clade-quartet tree corresponding triplets (bottom right). After filling this matrix with tree support scores inferred from all clade-quartets, the final support (S) for each possible rooted-clade tree with all clades included (RT1 to RT12; middle right) is calculated by summing up the individual rooted-triplet supports matching with this tree (top right). The best-supported rooted-clade tree is identified as the one with the highest sum of rooted-triplet support matching.

1.2 PhyQuart-mapping

PhyQuart-mapping focuses on four mutually exclusive predefined clades, each consisting of one or more species, defined as clade-quartet. Within this framework, there exists a collection of quartets formed by selecting one species from each of the four clades, named species-quartet. The relationships observed within these species-quartets represent the interrelationships among the corresponding clade-quartet, which is referred to as a clade-quartet-tree. The formula for the number of outgroup-containing clade-quartets, when choosing 3 clades out of n ingroup clades and the outgroup, is given by the combination formula:

$$\binom{n}{3} = \frac{n!}{3!(n-3)!} \tag{1.1}$$

PhyQuart-mapping summarizes individual PhyQuart analyses for all species-quartets of a clade-quartet, or alternatively, for a randomly selected subset of species-quartets if desired. Aggregate (mean and/or median) PhyQuart scores in PhyQuart-mapping offer a tree-independent evaluation of either non-trivial splits, such as internal branches derived from an existing tree, or relationships postulated based on other data, observations, or theories. It can also evaluate unspecified interrelationships among the four clades. In all scenarios, PhyQuart-mapping makes untested assumptions about clade membership, but significantly, it has the capacity to raise doubts regarding these assumptions.

High aggregate scores in this context indicate robust tree support and a strong signal, while low scores point to significant data conflict and the potential for systematic bias stemming from branch length heterogeneity, including effects like long-branch attraction or the attraction of short branches due to shared ancestral characteristics [2]. These factors can lead to erroneous splits and provide misleadingly high bootstrap values or other conventional branch support measures.

1.3 Supertree-approach (Icebreaker)

When a specified outgroup is provided, PhyQuart-mapping's aggregate support values can be utilized for outgroup-polarized assessments of relationships among more than four clades. In essence, PhyQuart-mapping transforms aggregate clade-quartet tree support values into a pairwise-compatibility support matrix, enabling the resolution of triplet polarization and representing intra-clade relationships. For example, an outgroup-polarized clade relationship $((C0, C1) \rightarrow (C2, C3))$ is deconstructed into two corresponding polarized triplets: $((R-C0) \rightarrow (C2, C3))$ and $((R-C1) \rightarrow (C2, C3))$. Here, the root is denoted by $R-$, and the clade-quartet support value assigned to $((C0, C1) \rightarrow (C2, C3))$ by PhyQuart-mapping is equally applied to both triplets (for further clarification, see Fig. 1.2).

It is important to note that both the outgroup $(C0)$ and the next related clade $(C1)$ are scored with respect to the root (R) since both clades are separated via the internal branch from $(C2, C3)$. This polarization of support is valid for both $C0$ (the outgroup) and $C1$ (the first split of the ingroup), as it reflects the separation of these clades from the more derived taxa $(C2, C3)$.

The pairwise-compatibility support matrix summarizes PhyQuart-mapping analysed tree scores for all clade-quartets within a potentially unlimited number of predefined, mutually ex-

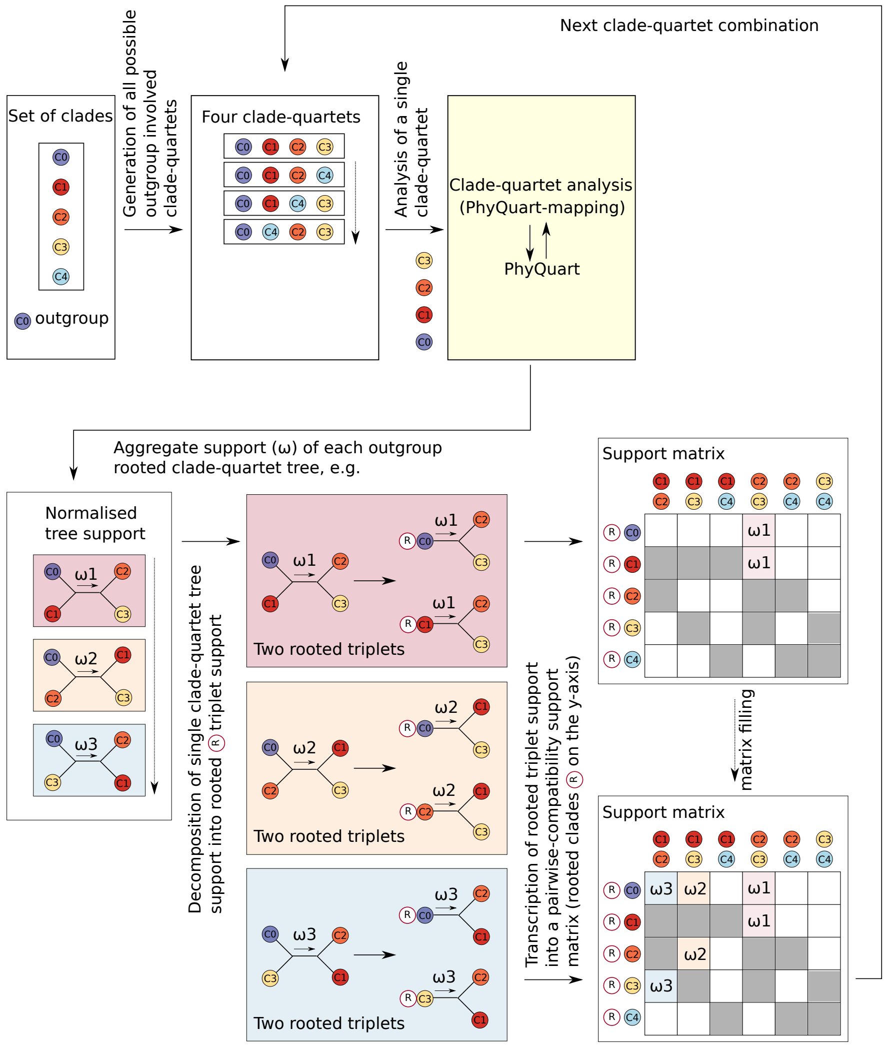

Figure 1.2: **Transfer of single clade-quartet tree support into a pairwise compatibility support matrix.** For a set of five clades ($C0$ (darkblue), $C1$ (darkred), $C2$ (red)), $C3$ (yellow), $C4$ (lightblue)) with clade $C0$ as the outgroup, four distinct clade-quartet combinations including the outgroup are possible. All combinations specific to the outgroup are analyzed individually using the PhyQuart-mapping process. The support identified for each of the three trees in a clade-quartet combination (ω_1 to ω_3) is then entered into a pairwise compatibility matrix, organized by rooted triplets (where the root is denoted by R). For instance, with the polarization along the internal branch of the clade-quartet tree $((O, A) \rightarrow (B, C))$ and PhyQuart scores ω_1, the initial clades ($C0$ and $C1$) are rooted as $R - C0$ and $R - C1$, and their support ω_1 is assigned to the compatibility matrix, where the rooted clades $R - C0$ and $R - C1$ appear on the y-axis and the derived clade pair $(C2, C3)$ on the x-axis. Grey boxes in the matrix indicate invalid combinations between rooted and derived clans, such as $R - C1$ and $(C1, C2)$. After conducting all clade-quartet analyses, each cell in the pairwise compatibility matrix represents the sum of single clade-quartet inferred support values (see Fig. 1.3).

clusive clades. All possible trees of a clade-quartet are evaluated. For a group of five clades, this involves the assessment of 12 distinct rooted-clade trees. To manage computation time, especially for a comprehensive tree search, it is recommended to limit the number of clades to ten at maximum (the number of species within each clade can vary significantly). The objective function involves the summation of support scores for all resolved triplets displayed in a candidate rooted-clade tree. Maximizing this function is equivalent to identifying a clique of triplets with the maximum weight (see Fig. 1.3).

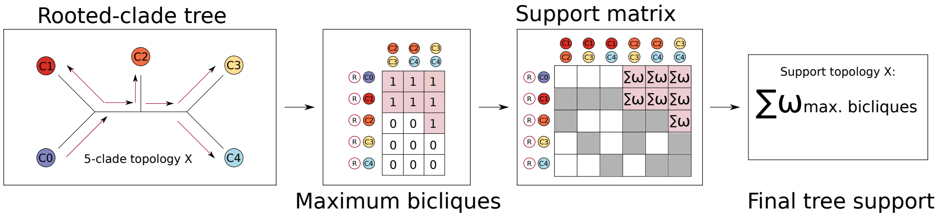

Figure 1.3: **Overview of rooted-clade tree evaluation using pairwise compatibility of rooted triplet support.** For a rooted-clade tree X, with clan $C0$ defined as the outgroup (circled in darkblue), the maximum biclique of compatible triplets consists of seven distinct triplet combinations (highlighted in red), involving a rooted $(R-)$ clade (y-axis) and a pair of derived clades (x-axis). The final support (maximum weight) for tree X is calculated as the sum of tree related rooted triplet support (ω) from the pairwise-compatibility support matrix $(\sum \omega_{max.bicliques})$.

1.4 Quartet Filtering

The quartet filtering process can occur on individual species-quartets using the quartet-related tree signal calculation of the PhyQuart algorithm (see description in Kück *et al.* (2017) [1]). Two different filter assumptions are implemented. The 'RISK' filter looks at the balance between potential shared derived characters and convergent signals, while the 'DIST' filter measures the actual differences in shared derived characters and helps identify which species-quartet tree has the least support conflict.

1.4.1 'RISK' Filter

The 'RISK' filter evaluates the ratio of PhyQuart signal that is convergently assigned (Nc) to the potentially apomorphic signal (Na) for each of the three possible topologies of a single species quartet (QT_1, QT_2, QT_3). To simplify terminology in the subsequent formulas and text passages, this Nc/Na ratio is abbreviated as $rNcNa$ in the following sections.

$$rNcNa_i = \frac{Nc_i}{Na_i}, \text{ for } i = QT_1, QT_2, QT_3 \quad (1.2)$$

The three individually evaluated tree ratios ($rNcNa_i$ for $i = QT_1, QT_2, QT_3$) of the corresponding species-quartet are compared to assess the distinctiveness of the apomorph signal (Na) relative to the convergent signal (Nc) among these three topologies. Both signals are estimated from the split-pattern distribution of tree supporting splits, either derived from the

original alignment observed pattern distribution (Na) or from maximum-likelihood estimations assuming one of the two other trees is correct (Nc) [1]. When $rNcNa$ for a species-quartet related tree is closer to one, it indicates a weaker apomorph signal for that specific tree. A value greater than one suggests that the convergently evolved tree signal dominates, implying that there's no significant apomorph signal left in the data for that particular tree.

Thus, the 'RISK' filter is designed under the theoretical assumption that the tree of a species-quartet with the lowest ratio of Nc to Na ($rNcNa_{best}$) is the most likely one (QT_{best}) for that particular species-quartet. This assumption is grounded in the idea that the best-supported tree should exhibit a higher number of apomorphies (Nap = $Na - Nc$ [1]) compared to alternative trees. The rationale behind favoring the species-quartet tree with the lowest $rNcNa_{best}$ lies in prioritizing signals indicating specific evolutionary traits unique to the best-supported relationship. A lower ratio suggests a greater abundance of apomorphies supporting the best tree, strengthening the argument for its accuracy in capturing the true evolutionary relationship. This prioritization aligns with the principle that a tree with a higher proportion of apomorphies is more likely to represent the correct phylogenetic signal in a species-quartet, contributing to a more reliable inference of evolutionary relationships within myriapods.

Following this assumption, the filter algorithm adheres to three key principles: i) The larger the difference in signal strength between QT_{best} and the other two trees of a species-quartet, the lower the conflict between this tree and the two other alternative relationship hypotheses. ii) The more pronounced the deviation of $rNcNa_{best}$ from 1 toward 0, the less vulnerable that tree is to the effects of convergently evolved signal (Nc). iii) Conversely, when $rNcNa_{best}$ is closer to 1, it becomes more susceptible to the influence of potentially undetected convergences, increasing the risk of an incorrect best tree signal.

To decide whether a quartet with the best rNcNa support should be rejected or retained, the value of $rNcNa_{best}$ is compared to an optimzed threshold 'L_{RISK}' (see section 1.4.3). If $rNcNa_{best}$ is below this threshold, it is assumed that QT_{best} is only minimally affected by convergent signal, and thus, this species-quartet is retained in the analysis.

$$rNcNa_{best} \leq L_{RISK} \implies \text{species-quartet retained in analysis} \tag{1.3}$$

On the contrary, if $rNcNa_{best}$ is greater than L_{RISK}, it is assumed that convergences have a substantial negative impact on QT_{best}, and that $rNcNa_{best}$ is in significant conflict with the $rNcNa$ values of the other two alternative trees. This species-quartet is rejected from the analysis.

$$rNcNa_{best} > L_{RISK} \implies \text{species-quartet rejected from analysis} \tag{1.4}$$

1.4.2 'DIST' Filter

The 'DIST' filter examines the support distance ($SD_{1,2}$) between the best and second-best species-quartet tree, effectively assessing the degree of signal conflict between them. A smaller support distance suggests higher conflict, while a larger distance indicates clearer phylogenetic signal supporting the best tree.

$$SD_{1,2} = \text{Support } QT_{best} - \text{Support } QT_{second} \tag{1.5}$$

The DIST filter evaluates absolute counts of apomorphic signals relative to conflicting signals. Specifically, it assesses the extent to which shared ancestral characters dominate over convergent signals. The smaller the support distance, the weaker the distinction between QT_{best} and QT_{second}, which implies ambiguous phylogenetic signal. To determine whether a species-quartet should be retained or excluded, $SD_{1,2}$ is compared to an optimized threshold L_{DIST}.

If $SD_{1,2} \leq L_{DIST}$, the species-quartet is rejected as the signal conflict is deemed too high.

$$SD_{1,2} \leq L_{DIST} \implies \text{species-quartet rejected from analysis} \tag{1.6}$$

If $SD_{1,2} > L_{DIST}$, the quartet is retained, as the signal supports a clear tree relationship.

$$SD_{1,2} > L_{DIST} \implies \text{species-quartet rejected from analysis} \tag{1.7}$$

By prioritizing quartets with strong support for one topology over the others, the DIST filter ensures that ambiguous signal conflicts are minimized in tree reconstruction.

1.4.3 Threshold Optimization

Regardless of whether combined or separate species-quartet filtering is employed, both filtering methods, 'RISK' and 'DIST', undergo individual threshold evaluations. Here, L_{RISK} functions as the upper threshold value, while L_{DIST} serves as the lower threshold. These thresholds are optimized to maintain a balance between the rejected and retained species-quartets using a common function, which is determined through an adapted uphill climbing algorithm, as detailed in the study by Misof *et al.* (2013) [3].

To select an optimal subset of remaining species-quartets for a clade-quartet, a range of thresholds is evaluated, starting from an initial value (L_{start}) and ending at a final value (L_{end}), with predefined increments. Therefore, rather than creating a gene presence-absence matrix with species on the y-axis and genes on the x-axis, the adopted uphill algorithm utilizes a presence-absence matrix (M) with species-quartets (n) on the x-axis and analyzed threshold values (L) on the y-axis. By default, for 'RISK,' the threshold values L_{start} and L_{end} are 0.7 and 1.0, respectively. In the case of 'DIST,' both of these values are 0.1 and 0.0. The default increment for threshold values in between is +0.1 for 'RISK' and -0.1 for 'DIST'. Consequently, under default settings, there are 31 single analyzed threshold values for 'RISK' ($N = 31$) and 11 for 'DIST' ($N = 11$). The total number of species-quartets (n) is determined by the quantity of clade-quartet individually analyzed species-quartets (For the complete count of quartets in each myriapod clade-quartet analysis, please see Table 2.7).

$$\text{Remaining species-quartets} = \begin{cases} 1 & \text{if species-quartet is retained,} \\ 0 & \text{if species-quartet is rejected.} \end{cases} \tag{1.8}$$

Thus, a matrix M of N thresholds and n species-quartets can be represented as a matrix with entries b_{ij}

$$M : b_{ij} = (1|0), \forall(thresholds : i : 1...N, quartets : j : 1...n) \tag{1.9}$$

We define the information content of a certain threshold value i, L_i as

$$L_i = \sum_{j}^{n} b_{ij}, \forall quartets : j : 1 \dots n \tag{1.10}$$

We define the information content, P, of a matrix M as

$$P(M) = \frac{\sum_{i=1}^{N} L_i}{N \times n} \tag{1.11}$$

with $P(M) \geq 0$.

We consider a threshold to be optimal when it combines a high information content, denoted as $P(M)$, and accommodates as many species-quartets for each clade-quartet as possible. Thus for each clade-quartet, the elimination of thresholds begins by removing the threshold with the lowest information content (L_i), which corresponds to the threshold with the highest number of clade-quartet related species-quartet rejections. The successive removal of L_i thresholds ultimately results in the creation of a 'trivial' threshold that retains as many species-quartets as possible. Thus, we implemented the optimality function introduced by Misof *et al.* [3]. This function utilizes a scaling factor α, with a default value of $\alpha = 3$, and λ, which denotes the size ratio between the reduced set M', consisting of the remaining thresholds, and the matrix M before the reduction step, covering all thresholds.

$$f(P) = \begin{cases} 1 - \left|\lambda - P^{\alpha \times (1-P)}\right| & \text{if } P < 1 \end{cases} \tag{1.12}$$

$$\lambda = \frac{N_{M'} \times n_{M'}}{N_M \times n_M} \tag{1.13}$$

As thresholds are progressively removed from the process, the value of P' steadily increases, while λ continuously decreases. The function $f(P')$ eventually reaches its maximum value of 1. When a scaling factor $\alpha = 2$ is employed, this maximum is attained at the intersection of P' and λ. In the case of $\alpha = 3$, the maximum is reached later in the process, indicating a preference for a optimal subset of remaining species-quartets with a higher P value, which corresponds to more remaining species-quartets. When $f(P') = 1$, the elimination process is concluded.

The computation time for this heuristic increases with the number of thresholds (N) and clade-quartet depending species-quartets (n). Consequently, it exhibits time efficiency with a complexity of $O(N + n)^2$. Additionally, the algorithm employs a deterministic approach for matrix reduction, ensuring reproducibility in the reduction process [3].

CHAPTER 2

Myriapoda Subgroup Relationships

2.1 SeaLion Parameter Specifications

Table 2.1 provides a comprehensive overview of the parameter specifications employed in two analyses: the investigation of myriapod subgroup relationships without specifying monophyletic Edafopoda (four ingroup clades), and the analysis incorporating Edafopoda (three ingroup clades with the clade Edafopoda merging Symphyla and Pauropoda).

For maximum-likelihood inference of expected convergence in the PhyQuart process [1], SeaLion uses the P4 package [4] to analyze each outgroup-polarized quartet tree. We optimized branch lengths and model parameters individually for each alternative quartet tree. The sequence evolution model chosen was the GTR+Γ+I model, with an α start-shape of rate heterogeneity set to 1.0 and a start-proportion of invariable sites (I) set at 0.3.

In single clade-quartet analyses (see section 2.2), all outgroup-polarized species-quartets are analyzed without any maximum limitations (we set the upper limit of single species-quartet analyses for each clade-quartet to 20,000, ensuring that this number exceeds the actual number of possible species-quartet combinations). After removing sites with ambiguous or incomplete (indel and missing) characters in species-quartet-related alignment positions, as described in Kück *et al.* (2017) [1], the minimum requirement for remaining site positions of was set at 5,000 bp. All analysed species-quartets met this criteria.

For Sealion, the median was selected as the measure of aggregate clade-quartet-tree related species-quartet-tree support due to its advantageous properties compared to the mean. The median is a robust choice, as it is less affected by outliers in the data, making it a reliable indicator of central tendency. It is particularly useful when dealing with skewed or non-normally distributed data, as it provides a more accurate representation of the central value. Additionally, the median is highly interpretable, making it a suitable choice for scenarios involving ordinal or categorical data. These advantages collectively make the median a preferred measure for our analysis of clade-quartet corresponding species-quartet-tree support.

We examined the second-codon-only dataset from Szucsich *et al.* (2020) [5] in both unfiltered and filtered species-quartet configurations. Species-quartet filtering in single clade-quartets was implemented using both 'RISK' as single filter and combined with the 'DIST' filter (see section 1.4) under default filter specifications (see Table 2.1). Further details about single parameter settings and their function are given in the SeaLion manual (https://github.com/PatrickKueck/SeaLion).

Table 2.1: SeaLion parameter print for both the clade analysis of myriapoda with and without Edafopoda.

SeaLion Option	Parameter
MSA infile:	STRICTnt2.fas
Clan infile:	clanfile_myriapoda.txt
Outgroup:	Out
Max. N quartets/4Clan:	20000
Min. site positions:	5000
Support-average:	median
SPD-infolder:	none
QUARTET-FILTER RISK	
RISK:	yes
Upper RISK limit:	1
Lower RISK limit:	0.7
RISK scale:	0.1
RISK alpha:	3
QUARTET-FILTER DIST	
DIST:	yes
Upper DIST limit:	0.1
Lower DIST limit:	0.0
DIST scale:	0.1
Lower DIST alpha:	3
P4-PARAMETER:	
Model (nu):	GTR
Start alpha:	1
Start pINV:	0.3
OUTPUT:	
Output-folder:	SeaLionGold_Myriapoda
Latex-table Print	yes
R-plotting	yes

2.2 Clade Assignments

We utilized SeaLion to analyze the 'STRICT' nucleotide dataset from Szucsich *et al.* (2020) [5], both with and without applying filters. For this analysis, we divided the dataset into five clades: the four myriapod subgroups and an outgroup containing representatives of Pancrustacea, Chelicerata, and Onychopohora (Table 2.2). Since each clade-quartet must incorporate the outgroup clade, this clade setting resulted in four distinct, rooted clade-quartets (Table 2.3).

Table 2.2: Reviewed clades and assigned codes for our myriapod subgroup relationship analysis without and with the definition of monophyletic Edafopoda.

Clade	Code	Analysis
Chilopoda	C	with & without Edafopoda
Diplopoda	D	with & without Edafopoda
Outgroup	O	with & without Edafopoda
Pauropoda	P	with Edafopoda
Symphyla	S	with Edafopoda
Symphyla & Pauropoda	X	without Edafopoda

Table 2.3: Reviewed clade-quartets and corresponding codes used in our clade analysis without monophyletic Edafopoda. Chilopoda: C; Diplopoda: D; Pauropoda: P; Symphyla: S.

Clade-Quartet					Code
Chilopoda	Diplura	Outgroup	Pauropoda		CDOP
Chilopoda	Diplura	Outgroup		Symphyla	CDOS
Chilopoda		Outgroup	Pauropoda	Symphyla	COPS
	Diplura	Outgroup	Pauropoda	Symphyla	DOPS

Additionally, we examined a single clade-quartet (Table 2.4) that consolidated Symphyla and Pauropoda into a single clade ’X’ (clade analysis with Edafopoda), a decision influenced by the results of our broader clade analysis without monophyletic Edafopoda. The analysis with monophyletic Edafopoda, uniting species of Symphyla and Pauropoda in a single clade, was aimed at providing further insights into the outgroup’s position and included an additional evaluation of the relationship between Diplopoda and Chilopoda due to a reduced number of clade-quartets and a various composition of available species-quartets.

Table 2.4: Examined clade-quartet (4C) and assigned code, designating Symphyla and Pauropoda as a single clade named Edafopoda (code as X).

Clade-Quartet				Code
Chilopoda	Diplura	Outgroup	Pauropoda & Symphyla	CDOX

2.3 Clade Analysis without Edafopoda

2.3.1 Filter Optimization

Optimal thresholds for the RISK and DIST filters are individually determined for each of the four clade-quartets in our myriapod subgroup analysis without Edafopoda. This optimization, affecting the number of remaining species-quartets (Table 2.5), aims to achieve a balance between support and conflict for phylogenetic hypotheses. We initiated the filter optimization using the default parameters of SeaLion. The RISK filter started with a threshold of 0.7, incrementing in steps of 0.1 until reaching the upper threshold limit of 1. For the DIST filter, we set the start value to 0.1, reducing it in steps of 0.1 (see threshold descriptions in section 1.4.3). Changes of the threshold depending information content of remaining species-quartets in each optimization step are shown for the 'RISK' filter in Fig. 2.1 while number of remaining species-quartets for each analysed threshold are shown in Fig. 2.2. For the 'DIST' filter see Fig. 2.3 and 2.4, respectively.

Table 2.5: The total number of individual species-quartet analyses (Nq), encompassing both unfiltered and filtered datasets using the 'RISK' filter alone and in combination with the 'DIST' filter, along with the corresponding optimized threshold values (L_{RISK} and L_{DIST}).

Clade-Quartet	Nq 'Unfiltered'	L_{RISK}	Nq 'RISK'	L_{DIST}	Nq 'RISK+DIST'
CDOP	4.640	0.77	1.495	0.1	1.288
CDOS	13.920	0.77	3.780	0.1	3.388
COPS	1.392	0.76	507	0.1	492
DOPS	870	0.76	217	0.1	209

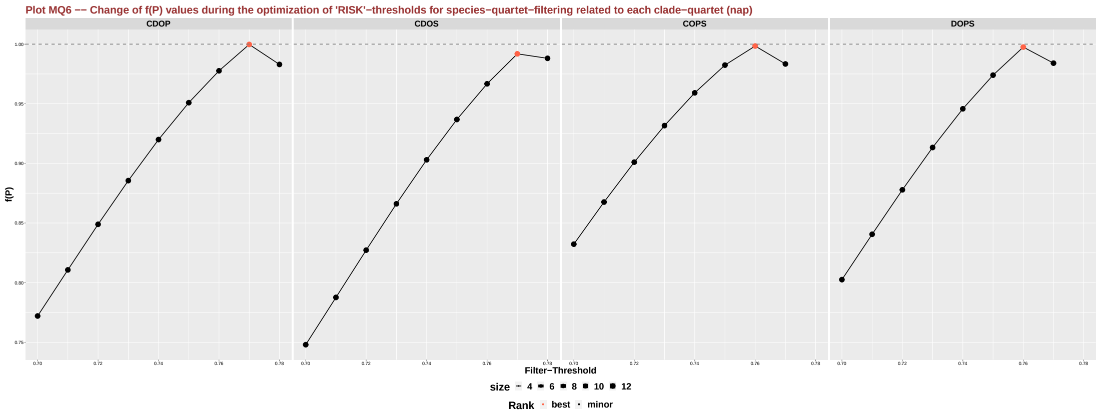

Figure 2.1: Optimized **'RISK' threshold** values (Tl, x-axis) for each clade-quartet as a function of the signal content for different trees in remaining species-quartets ($f(P)$), printed by SeaLion. The best threshold is highlighted in red.

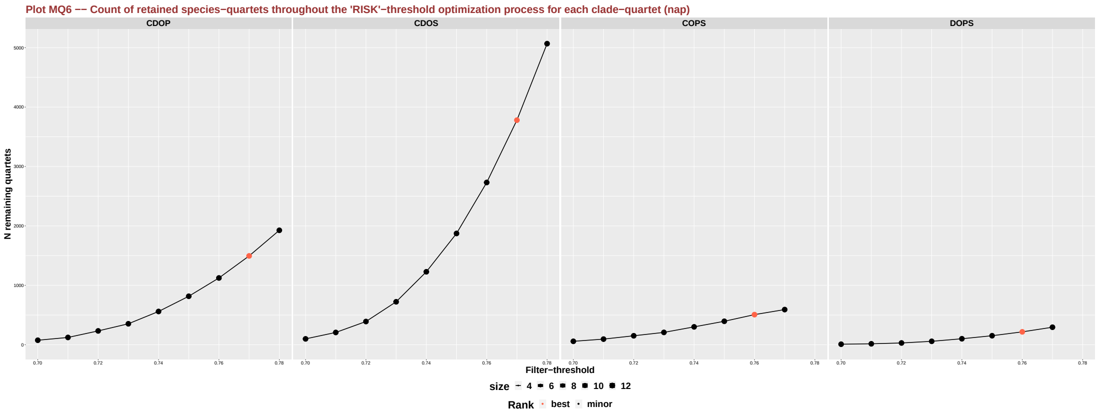

Figure 2.2: Remaining quartets (y-axis) corresponding to **'RISK' threshold** values (Tl, x-axis) for each clade-quartet, printed by SeaLion. The best threshold is highlighted in red.

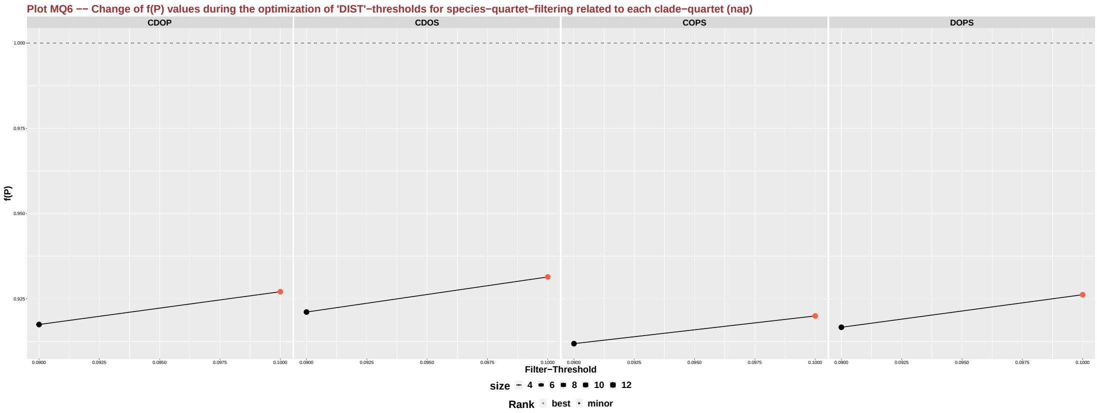

Figure 2.3: Optimized **'DIST' threshold** values (Tl, x-axis) for each clade-quartet as a function of the signal content for different trees in remaining species-quartets ($f(P)$), printed by SeaLion. The best threshold is highlighted in red.

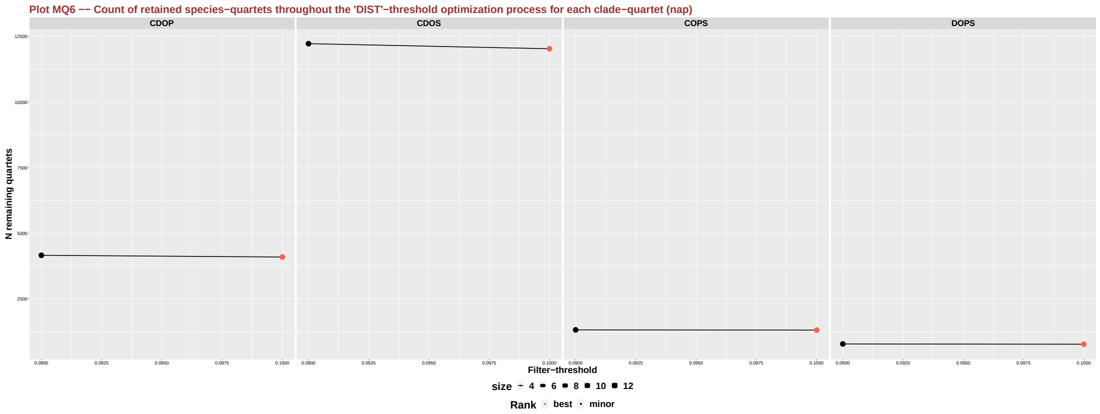

Figure 2.4: Remaining quartets (y-axis) corresponding to **'DIST' threshold** values (TI, x-axis) for each clade-quartet, printed by SeaLion. The best threshold is highlighted in red.

2.3.2 Clade-Quartet Related Tree Support

Median tree support for single clade-quartet relationships for the unfiltered and filtered ('RISK' and 'RISK+DIST') analysis of myriapod subgroup relationships without Edafopoda, inferred from clade-quartet corresponding species-quartets is shown in Tab. 2.6. Individual support values derived for each clade-quartet tree are standardized relative to each other, resulting in a support spectrum ranging from zero (indicating no support) to 1 (representing perfect support). The best supported clade-quartet trees with the respective counts of support underlying species-quartets are once more extracted in Table 2.7.

Table 2.6: Standardized median clade-quartet (4C) tree support inferred from both unfiltered and filtered ('RISK' and 'RISK+DIST') species-quartet analyses, printed by SeaLion.

4C	Filter	4C Tree	Tree Code	Aggregate	Support
CDOP	unfiltered	(Out,(Chil,(Dipl,Paur)))	QT1	median	0.27
CDOP	unfiltered	(Out,(Dipl,(Chil,Paur)))	QT2	median	0.16
CDOP	unfiltered	(Out,(Paur,(Chil,Dipl)))	QT3	median	0.56
CDOP	RISK	(Out,(Chil,(Dipl,Paur)))	QT1	median	0.78
CDOP	RISK	(Out,(Dipl,(Chil,Paur)))	QT2	median	0.22
CDOP	RISK	(Out,(Paur,(Chil,Dipl)))	QT3	median	0.00
CDOP	RISK+DIST	(Out,(Chil,(Dipl,Paur)))	QT1	median	0.79
CDOP	RISK+DIST	(Out,(Dipl,(Chil,Paur)))	QT2	median	0.21
CDOP	RISK+DIST	(Out,(Paur,(Chil,Dipl)))	QT3	median	0.00
CDOS	unfiltered	(Out,(Chil,(Dipl,Symp)))	QT1	median	0.42
CDOS	unfiltered	(Out,(Dipl,(Chil,Symp)))	QT2	median	0.25
CDOS	unfiltered	(Out,(Symp,(Chil,Dipl)))	QT3	median	0.34
CDOS	RISK	(Out,(Chil,(Dipl,Symp)))	QT1	median	0.82

Continues on next page(s).

CDOS	RISK	(Out,(Dipl,(Chil,Symp)))	QT2	median	0.18
CDOS	RISK	(Out,(Symp,(Chil,Dipl)))	QT3	median	0.00
CDOS	RISK+DIST	(Out,(Chil,(Dipl,Symp)))	QT1	median	0.84
CDOS	RISK+DIST	(Out,(Dipl,(Chil,Symp)))	QT2	median	0.16
CDOS	RISK+DIST	(Out,(Symp,(Chil,Dipl)))	QT3	median	0.00
COPS	unfiltered	(Out,(Chil,(Paur,Symp)))	QT1	median	0.62
COPS	unfiltered	(Out,(Paur,(Chil,Symp)))	QT2	median	0.38
COPS	unfiltered	(Out,(Symp,(Chil,Paur)))	QT3	median	0.00
COPS	RISK	(Out,(Chil,(Paur,Symp)))	QT1	median	0.91
COPS	RISK	(Out,(Paur,(Chil,Symp)))	QT2	median	0.09
COPS	RISK	(Out,(Symp,(Chil,Paur)))	QT3	median	0.00
COPS	RISK+DIST	(Out,(Chil,(Paur,Symp)))	QT1	median	0.92
COPS	RISK+DIST	(Out,(Paur,(Chil,Symp)))	QT2	median	0.08
COPS	RISK+DIST	(Out,(Symp,(Chil,Paur)))	QT3	median	0.00
DOPS	unfiltered	(Out,(Dipl,(Paur,Symp)))	QT1	median	0.45
DOPS	unfiltered	(Out,(Paur,(Dipl,Symp)))	QT2	median	0.52
DOPS	unfiltered	(Out,(Symp,(Dipl,Paur)))	QT3	median	0.04
DOPS	RISK	(Out,(Dipl,(Paur,Symp)))	QT1	median	0.87
DOPS	RISK	(Out,(Paur,(Dipl,Symp)))	QT2	median	0.11
DOPS	RISK	(Out,(Symp,(Dipl,Paur)))	QT3	median	0.02
DOPS	RISK+DIST	(Out,(Dipl,(Paur,Symp)))	QT1	median	0.88
DOPS	RISK+DIST	(Out,(Paur,(Dipl,Symp)))	QT2	median	0.11
DOPS	RISK+DIST	(Out,(Symp,(Dipl,Paur)))	QT3	median	0.01

End of table 2.6

Table 2.7: Standardized median support of analysed species-quartets (Nq) for the best clade-quartet (4C) tree in both unfiltered and filtered ('RISK' and 'RISK+DIST') quartet analyses, printed by SeaLion.

4C	Filter	Best 4C Tree	Tree Code	Aggregate	Support	Nq
CDOP	unfiltered	(Out,(Paur,(Chil,Dipl)))	QT3	median	0.56	4640
CDOP	RISK	(Out,(Chil,(Dipl,Paur)))	QT1	median	0.78	1495
CDOP	RISK+DIST	(Out,(Chil,(Dipl,Paur)))	QT1	median	0.79	1288
CDOS	unfiltered	(Out,(Chil,(Dipl,Symp)))	QT1	median	0.42	13920
CDOS	RISK	(Out,(Chil,(Dipl,Symp)))	QT1	median	0.82	3780
CDOS	RISK+DIST	(Out,(Chil,(Dipl,Symp)))	QT1	median	0.84	3388
COPS	unfiltered	(Out,(Chil,(Paur,Symp)))	QT1	median	0.62	1392
COPS	RISK	(Out,(Chil,(Paur,Symp)))	QT1	median	0.91	507
COPS	RISK+DIST	(Out,(Chil,(Paur,Symp)))	QT1	median	0.92	492
DOPS	unfiltered	(Out,(Paur,(Dipl,Symp)))	QT2	median	0.52	870
DOPS	RISK	(Out,(Dipl,(Paur,Symp)))	QT1	median	0.87	217
DOPS	RISK+DIST	(Out,(Dipl,(Paur,Symp)))	QT1	median	0.88	209

2.3.3 Species Related Quartet Participations

A summarized overview of single species related quartet contributions in single clade-quartets of the unfiltered and filtered ('RISK', 'RISK+DIST') analyses are listed in Tab. 2.8. The collective count of single species contributions across all analyzed clade-quartets, following species-quartet filtering, is depicted for the 'RISK' filter in Fig. 2.5 and for the 'DIST' filter in Fig. 2.6.

Table 2.8: Number of species participations in species-quartets of both unfiltered and filtered ('RISK' and 'RISK+DIST') clade-quartet (4C) analyses, printed by SeaLion.

Species	Clade	4C	Unfiltered	RISK	RISK+DIST
Acerentomon_maius	O	CDOP	160	12	8
Acerentomon_maius	O	CDOS	480	27	22
Acerentomon_maius	O	COPS	48	0	0
Acerentomon_maius	O	DOPS	30	0	0
Acopauropus_ornatus	P	CDOP	4640	1495	1288
Acopauropus_ornatus	P	COPS	1392	507	492
Acopauropus_ornatus	P	DOPS	870	217	209
Anaspides_tasmaniae	O	CDOP	160	16	16
Anaspides_tasmaniae	O	CDOS	480	22	18
Anaspides_tasmaniae	O	COPS	48	4	4
Anaspides_tasmaniae	O	DOPS	30	0	0
Anurida_maritima	O	CDOP	160	0	0
Anurida_maritima	O	CDOS	480	1	1
Anurida_maritima	O	COPS	48	0	0
Anurida_maritima	O	DOPS	30	0	0
Araneus_diadematus	O	CDOP	160	79	60
Araneus_diadematus	O	CDOS	480	133	105
Araneus_diadematus	O	COPS	48	27	27
Araneus_diadematus	O	DOPS	30	7	7
Archegozetes_longisetosus	O	CDOP	160	0	0
Archegozetes_longisetosus	O	CDOS	480	1	0
Archegozetes_longisetosus	O	COPS	48	0	0
Archegozetes_longisetosus	O	DOPS	30	0	0
Atelura_formicaria	O	CDOP	160	105	88
Atelura_formicaria	O	CDOS	480	253	231
Atelura_formicaria	O	COPS	48	35	34
Atelura_formicaria	O	DOPS	30	11	10
Callipus_foetidissimus	D	CDOP	464	117	104
Callipus_foetidissimus	D	CDOS	1392	343	290
Callipus_foetidissimus	D	DOPS	87	36	36
Cinopodes_flavidus	C	CDOP	290	60	51
Cinopodes_flavidus	C	CDOS	870	133	112
Cinopodes_flavidus	C	COPS	87	19	19

Continues on next page(s).

Craspedosoma_sp	D	CDOP	464	130	117
Craspedosoma_sp	D	CDOS	1392	372	358
Craspedosoma_sp	D	DOPS	87	29	27
Craterostigmus_tasmanianus	C	CDOP	290	93	83
Craterostigmus_tasmanianus	C	CDOS	870	231	202
Craterostigmus_tasmanianus	C	COPS	87	35	35
Cryptops_anomalans	C	CDOP	290	92	73
Cryptops_anomalans	C	CDOS	870	168	160
Cryptops_anomalans	C	COPS	87	31	27
Cryptops_hortensis_F2014	C	CDOP	290	85	69
Cryptops_hortensis_F2014	C	CDOS	870	183	166
Cryptops_hortensis_F2014	C	COPS	87	27	26
Damon_diadema	O	CDOP	160	159	150
Damon_diadema	O	CDOS	480	455	401
Damon_diadema	O	COPS	48	48	48
Damon_diadema	O	DOPS	30	29	27
Daphnia_pulex	O	CDOP	160	3	3
Daphnia_pulex	O	CDOS	480	13	12
Daphnia_pulex	O	COPS	48	0	0
Daphnia_pulex	O	DOPS	30	0	0
EDANI	O	CDOP	160	45	41
EDANI	O	CDOS	480	87	83
EDANI	O	COPS	48	14	14
EDANI	O	DOPS	30	2	2
Egaenus_convexus	O	CDOP	160	145	128
Egaenus_convexus	O	CDOS	480	335	279
Egaenus_convexus	O	COPS	48	44	42
Egaenus_convexus	O	DOPS	30	21	21
Essigella_californica	O	CDOP	160	0	0
Essigella_californica	O	CDOS	480	0	0
Essigella_californica	O	COPS	48	0	0
Essigella_californica	O	DOPS	30	0	0
EubranCpus_grubii	O	CDOP	160	2	1
EubranCpus_grubii	O	CDOS	480	9	9
EubranCpus_grubii	O	COPS	48	3	0
EubranCpus_grubii	O	DOPS	30	0	0
Eudigraphis_takakuwai	D	CDOP	464	256	240
Eudigraphis_takakuwai	D	CDOS	1392	546	493
Eudigraphis_takakuwai	D	DOPS	87	14	13
Eupolybothrus_cavernicolus_sp	C	CDOP	290	133	112
Eupolybothrus_cavernicolus_sp	C	CDOS	870	360	323
Eupolybothrus_cavernicolus_sp	C	COPS	87	44	43
Eupolybothrus_cf_fasciatus	C	CDOP	290	151	137
Eupolybothrus_cf_fasciatus	C	CDOS	870	453	423
Eupolybothrus_cf_fasciatus	C	COPS	87	44	42

Continues on next page(s).

Eupolybothrus_tridentinus	C	CDOP	290	136	117
Eupolybothrus_tridentinus	C	CDOS	870	362	325
Eupolybothrus_tridentinus	C	COPS	87	44	40
Euscorpius_sicanus	O	CDOP	160	151	126
Euscorpius_sicanus	O	CDOS	480	364	345
Euscorpius_sicanus	O	COPS	48	47	47
Euscorpius_sicanus	O	DOPS	30	26	26
Glomeridella_minima	D	CDOP	464	108	90
Glomeridella_minima	D	CDOS	1392	372	335
Glomeridella_minima	D	DOPS	87	17	16
Hanseniella_nivea	S	CDOS	4640	1007	900
Hanseniella_nivea	S	COPS	464	163	157
Hanseniella_nivea	S	DOPS	290	59	58
Hanseniella_sp	S	CDOS	4640	1511	1381
Hanseniella_sp	S	COPS	464	167	158
Hanseniella_sp	S	DOPS	290	67	61
Haploglomeris_multistriata	D	CDOP	464	122	92
Haploglomeris_multistriata	D	CDOS	1392	389	358
Haploglomeris_multistriata	D	DOPS	87	19	17
Hemidiaptomus_amblyodon	O	CDOP	160	0	0
Hemidiaptomus_amblyodon	O	CDOS	480	0	0
Hemidiaptomus_amblyodon	O	COPS	48	0	0
Hemidiaptomus_amblyodon	O	DOPS	30	0	0
Henia_illyrica	C	CDOP	290	54	46
Henia_illyrica	C	CDOS	870	133	116
Henia_illyrica	C	COPS	87	14	14
Himantarium_gabrielis	C	CDOP	290	80	68
Himantarium_gabrielis	C	CDOS	870	176	164
Himantarium_gabrielis	C	COPS	87	35	35
Ixodes_scapularis	O	CDOP	160	23	23
Ixodes_scapularis	O	CDOS	480	26	26
Ixodes_scapularis	O	COPS	48	2	2
Ixodes_scapularis	O	DOPS	30	5	5
LFUL	O	CDOP	160	69	54
LFUL	O	CDOS	480	208	201
LFUL	O	COPS	48	32	32
LFUL	O	DOPS	30	5	4
Lithobius_forficatus	C	CDOP	290	123	103
Lithobius_forficatus	C	CDOS	870	339	298
Lithobius_forficatus	C	COPS	87	41	40
MaCis_hrabei	O	CDOP	160	87	78
MaCis_hrabei	O	CDOS	480	226	190
MaCis_hrabei	O	COPS	48	26	26
MaCis_hrabei	O	DOPS	30	19	18
Nebalia_bipes	O	CDOP	160	0	0

Continues on next page(s).

Nebalia_bipes	O	CDOS	480	0	0
Nebalia_bipes	O	COPS	48	0	0
Nebalia_bipes	O	DOPS	30	0	0
Nymphon_gracile	O	CDOP	160	58	56
Nymphon_gracile	O	CDOS	480	160	154
Nymphon_gracile	O	COPS	48	33	33
Nymphon_gracile	O	DOPS	30	13	13
Occasjapyx_japonicus_neu	O	CDOP	160	51	33
Occasjapyx_japonicus_neu	O	CDOS	480	231	188
Occasjapyx_japonicus_neu	O	COPS	48	24	19
Occasjapyx_japonicus_neu	O	DOPS	30	5	5
Ommatoiulus_sabulosus	D	CDOP	464	146	117
Ommatoiulus_sabulosus	D	CDOS	1392	343	291
Ommatoiulus_sabulosus	D	DOPS	87	32	31
Peripatoides_novaezealandiae	O	CDOP	160	121	115
Peripatoides_novaezealandiae	O	CDOS	480	304	296
Peripatoides_novaezealandiae	O	COPS	48	47	47
Peripatoides_novaezealandiae	O	DOPS	30	19	19
Peripatopsis_capensis_F2014	O	CDOP	160	145	134
Peripatopsis_capensis_F2014	O	CDOS	480	360	352
Peripatopsis_capensis_F2014	O	COPS	48	48	48
Peripatopsis_capensis_F2014	O	DOPS	30	27	26
Periplaneta_americana	O	CDOP	160	102	78
Periplaneta_americana	O	CDOS	480	250	197
Periplaneta_americana	O	COPS	48	35	33
Periplaneta_americana	O	DOPS	30	13	12
Pogonognathellus_sp	O	CDOP	160	0	0
Pogonognathellus_sp	O	CDOS	480	1	1
Pogonognathellus_sp	O	COPS	48	0	0
Pogonognathellus_sp	O	DOPS	30	0	0
Polydesmus_complanatus	D	CDOP	464	189	157
Polydesmus_complanatus	D	CDOS	1392	525	487
Polydesmus_complanatus	D	DOPS	87	15	15
Polyxenus_lagurus	D	CDOP	464	199	176
Polyxenus_lagurus	D	CDOS	1392	459	400
Polyxenus_lagurus	D	DOPS	87	10	9
Polyzonium_germanicum	D	CDOP	464	122	96
Polyzonium_germanicum	D	CDOS	1392	252	216
Polyzonium_germanicum	D	DOPS	87	18	18
Schendyla_cf_carniolensis	C	CDOP	290	61	49
Schendyla_cf_carniolensis	C	CDOS	870	140	124
Schendyla_cf_carniolensis	C	COPS	87	19	19
Scolopendra_cingulata	C	CDOP	290	102	85
Scolopendra_cingulata	C	CDOS	870	268	239
Scolopendra_cingulata	C	COPS	87	40	40

Continues on next page(s).

Scolopocryptops_rubiginosus	C	CDOP	290	75	69
Scolopocryptops_rubiginosus	C	CDOS	870	236	209
Scolopocryptops_rubiginosus	C	COPS	87	38	37
Scutigera_coleoptrata	C	CDOP	290	126	113
Scutigera_coleoptrata	C	CDOS	870	376	325
Scutigera_coleoptrata	C	COPS	87	46	45
Strigamia_acuminata	C	CDOP	290	58	52
Strigamia_acuminata	C	CDOS	870	124	111
Strigamia_acuminata	C	COPS	87	16	16
Strigamia_maritima	C	CDOP	290	66	61
Strigamia_maritima	C	CDOS	870	98	91
Strigamia_maritima	C	COPS	87	14	14
Subilla_sp	O	CDOP	160	15	11
Subilla_sp	O	CDOS	480	22	19
Subilla_sp	O	COPS	48	0	0
Subilla_sp	O	DOPS	30	0	0
Symphylella_sp	S	CDOS	4640	1262	1107
Symphylella_sp	S	COPS	464	177	177
Symphylella_sp	S	DOPS	290	91	90
Thalassisobates_littoralis	D	CDOP	464	106	99
Thalassisobates_littoralis	D	CDOS	1392	197	160
Thalassisobates_littoralis	D	DOPS	87	27	27
Tisbe_furcata	O	CDOP	160	0	0
Tisbe_furcata	O	CDOS	480	0	0
Tisbe_furcata	O	COPS	48	0	0
Tisbe_furcata	O	DOPS	30	0	0
Triops_cancriformis	O	CDOP	160	12	8
Triops_cancriformis	O	CDOS	480	28	26
Triops_cancriformis	O	COPS	48	1	0
Triops_cancriformis	O	DOPS	30	0	0
Vargula_hilgendorfii	O	CDOP	160	13	8
Vargula_hilgendorfii	O	CDOS	480	34	28
Vargula_hilgendorfii	O	COPS	48	4	3
Vargula_hilgendorfii	O	DOPS	30	0	0
Xibalbanus_tulumensis	O	CDOP	160	82	69
Xibalbanus_tulumensis	O	CDOS	480	230	204
Xibalbanus_tulumensis	O	COPS	48	33	33
Xibalbanus_tulumensis	O	DOPS	30	15	14

End of table 2.8

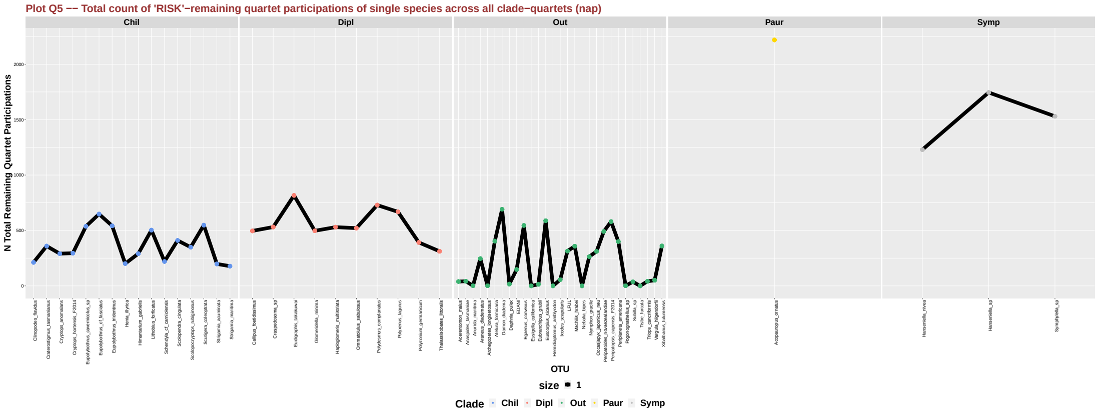

Figure 2.5: **’RISK’ filtering**: Overall count of species (x-axis) related quartet participations (y-axis), remaining across all clade-quartets, printed by SeaLion.

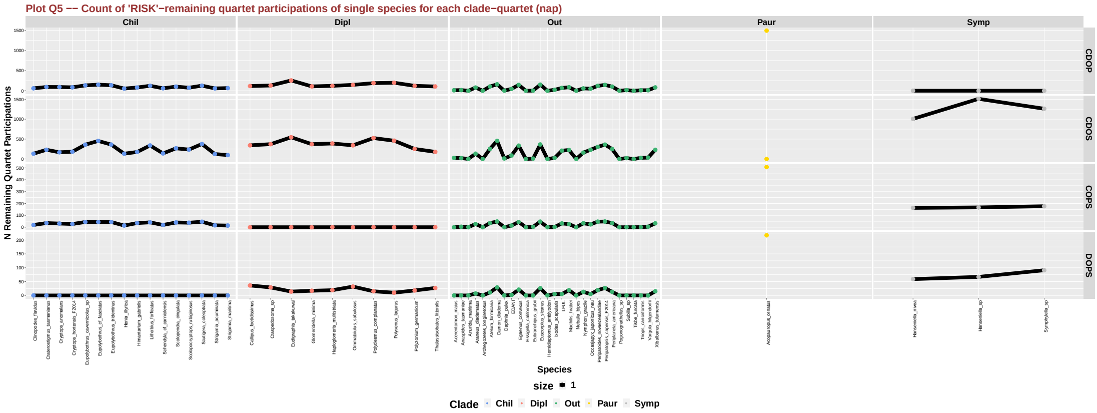

Figure 2.6: **’DIST’ filtering**: Overall count of species (x-axis) related quartet participations (y-axis), remaining across all clade-quartets, printed by SeaLion.

2.3.4 Species Related Clade-Quartet Tree Support Contribution

In addition to evaluating the median support for each clade-quartet tree derived from all contributing species-quartets, SeaLion also examines the species-specific contributions to single clade-quartet tree support exclusively in quartets related to individual species. The support contributions related to individual species are represented by the median tree support for each clade-quartet, with tree support values normalized to each other. Unfiltered species-related support contributions for each clade-quartet tree are illustrated in Fig. 2.7. Species support contributions following ’RISK’ and ’RISK+DIST’ species-quartet filtering analyses are presented in Fig. 2.8 and Fig. 2.9, respectively.

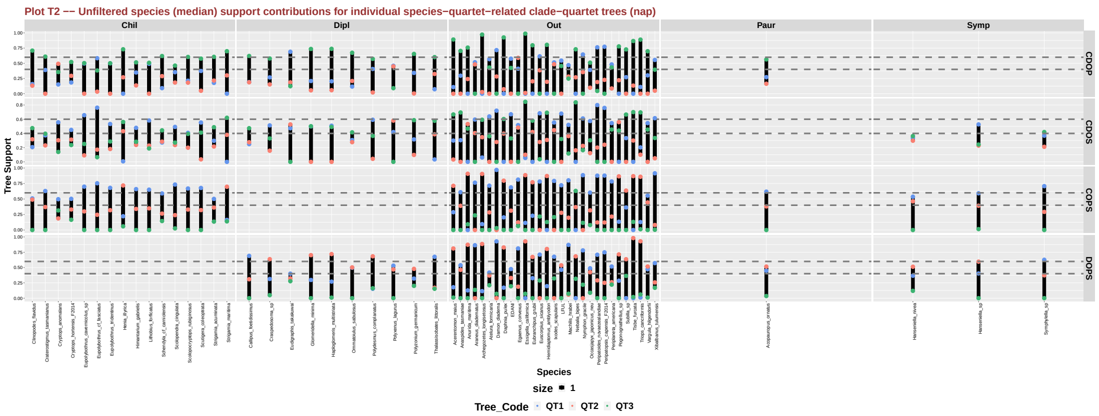

Figure 2.7: Support contributions from individual species (x-axis) in **unfiltered species-quartets** are depicted for each of the three trees (QT1, QT2, QT3; for corresponding newick string see Table 2.9) in a clade-quartet (y-axis, right), printed by SeaLion. Black vertical lines emphasize median support distances between different trees within the same clade-quartet.

Table 2.9: Designated tree codes, applicable to every clade-quartet (4C), printed by SeaLion.

4C	QT1	QT2	QT3
CDOP	(Out,(Chil,(Dipl,Paur)))	(Out,(Dipl,(Chil,Paur)))	(Out,(Paur,(Chil,Dipl)))
CDOS	(Out,(Chil,(Dipl,Symp)))	(Out,(Dipl,(Chil,Symp)))	(Out,(Symp,(Chil,Dipl)))
COPS	(Out,(Chil,(Paur,Symp)))	(Out,(Paur,(Chil,Symp)))	(Out,(Symp,(Chil,Paur)))
DOPS	(Out,(Dipl,(Paur,Symp)))	(Out,(Paur,(Dipl,Symp)))	(Out,(Symp,(Dipl,Paur)))

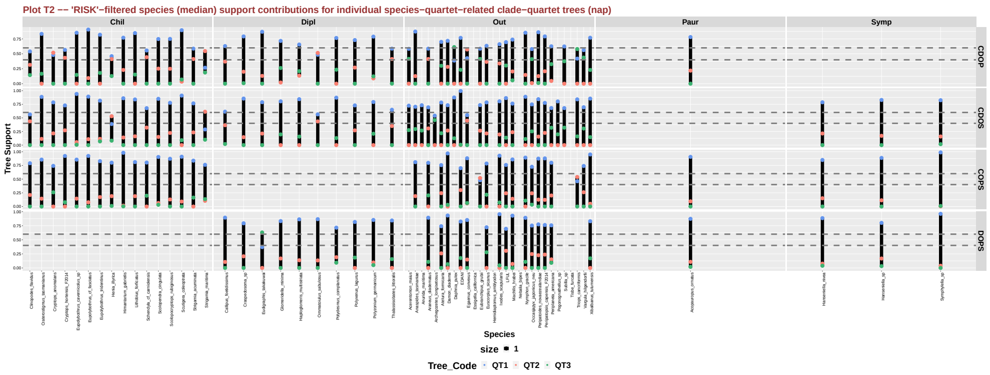

Figure 2.8: Support contributions from individual species (x-axis) in **'RISK' filtered species-quartets** are depicted for each of the three trees (QT1, QT2, QT3; for corresponding newick string see Table 2.9) in a clade-quartet (y-axis, right), printed by SeaLion. Black vertical lines emphasize median support distances between different trees within the same clade-quartet.

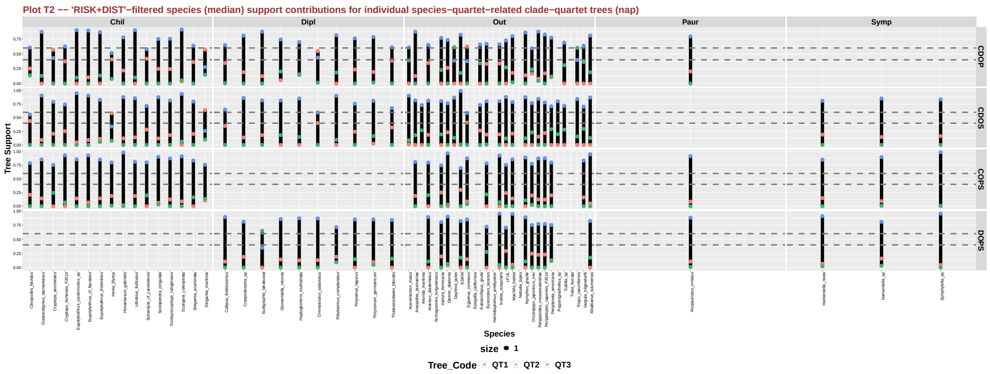

Figure 2.9: Support contributions from individual species (x-axis) in **'RISK+DIST' filtered species-quartets** are depicted for each of the three trees (QT1, QT2, QT3; for corresponding newick string see Table 2.9) in a clade-quartet (y-axis, right), printed by SeaLion. Black vertical lines emphasize median support distances between different trees within the same clade-quartet.

2.4 Clade Analysis with Edafopoda

2.4.1 Filter Optimization

Optimal thresholds for the RISK and DIST filters are determined individually for the single clade-quartet (CDOX) in our myriapod subgroup analysis with Edafopoda, following the procedure outlined in our analysis without Edafopoda (see section 2.3.1). Changes of the threshold depending information content of remaining species-quartets in each optimization step are shown for the 'RISK' filter in Fig. 2.10 while number of remaining species-quartets for each analysed threshold are shown in Fig. 2.11. For the 'DIST' filter see Fig. 2.12 and 2.13, respectively. The threshold impact to 'RISK' filtered tree support is given in Fig. 2.14.

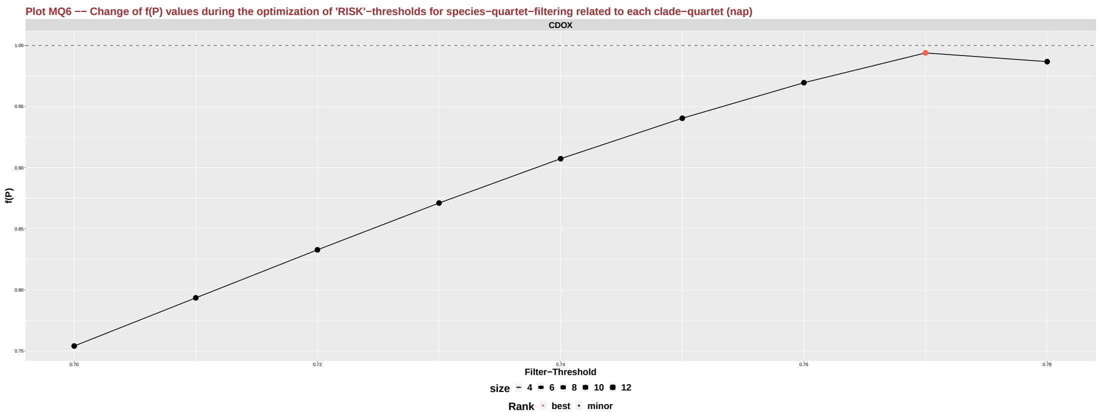

Figure 2.10: Optimized **'RISK' threshold** values (Tl, x-axis) for each clade-quartet as a function of the signal content for different trees in remaining species-quartets ($f(P)$), printed by SeaLion. The best threshold is highlighted in red.

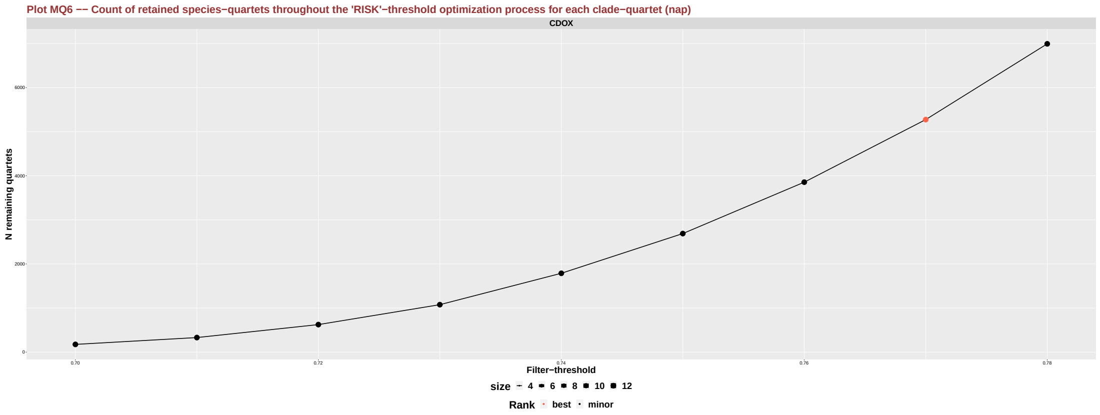

Figure 2.11: Remaining quartets (y-axis) corresponding to **'RISK' threshold** values (Tl, x-axis) for each clade-quartet, printed by SeaLion. The best threshold is highlighted in red.

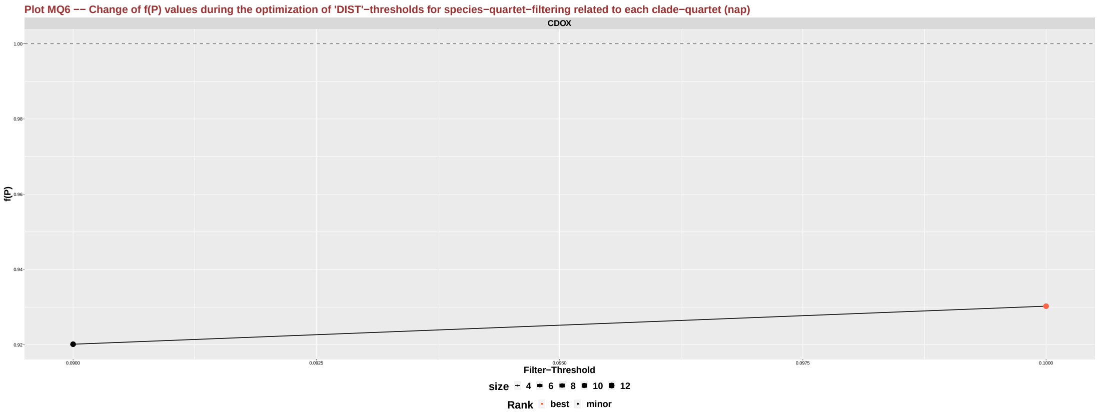

Figure 2.12: Optimized **'DIST' threshold** values (Tl, x-axis) for each clade-quartet as a function of the signal content for different trees in remaining species-quartets ($f(P)$), printed by SeaLion. The best threshold is highlighted in red.

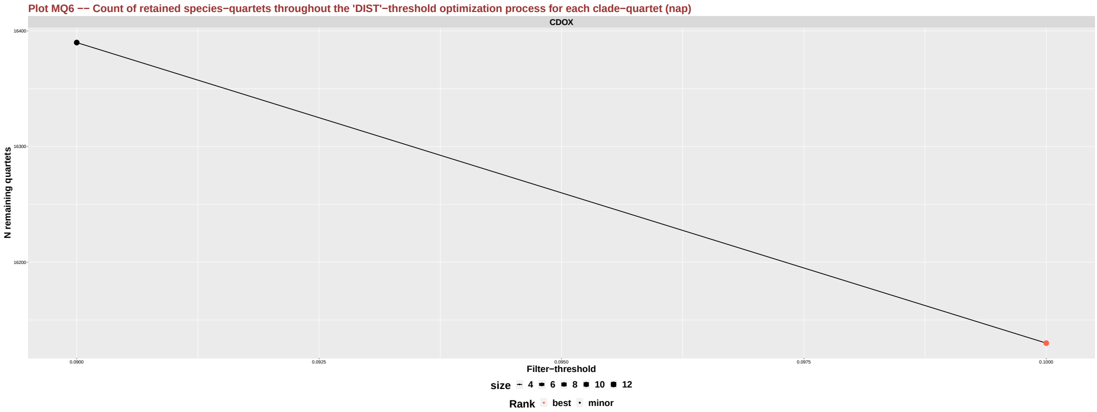

Figure 2.13: Remaining quartets (y-axis) corresponding to **'DIST' threshold** values (Tl, x-axis) for each clade-quartet, printed by SeaLion. The best threshold is highlighted in red.

2.4.2 RISK Filter Impact on Species-Quartets

The aim of the 'RISK' filter is to keep a balance between rejected and remaining quartets by preserving as many best trees (blue dots) of high information content, expressed by a low Nc to Na proportion, as possible without loosing too many quartets across a clade combination (see detailed description in section 1.4). The impact of tree support signal after 'RISK' filtering species-quartets for the clade-quartet with Edafopoda (CDOX) is shown in Fig. 2.14.

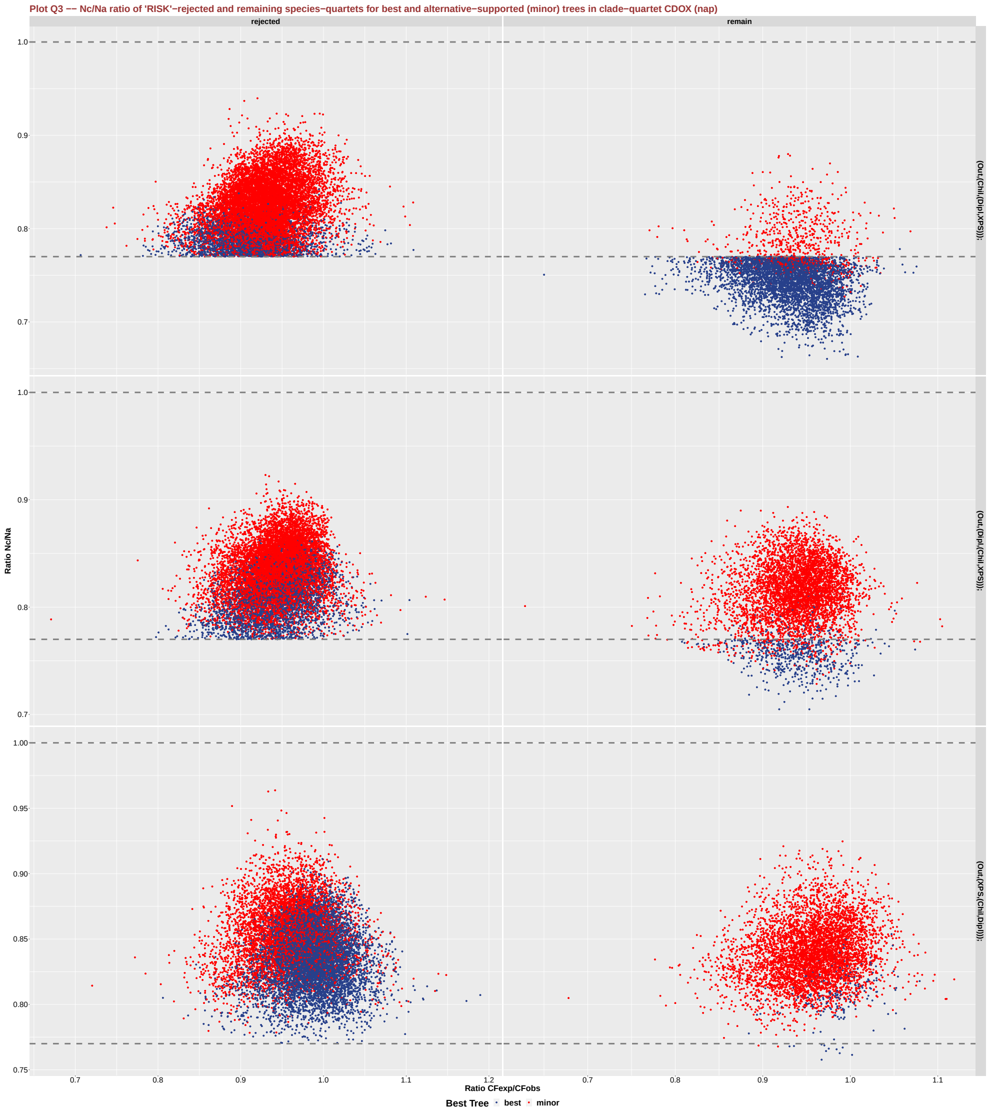

Figure 2.14: Remaining (right column) and rejected (left column) species-quartets with tree related (y-axis right) support expressed as ratios of potentially convergent ('Nc') to apomorphic ('Na') character states (y-axis left), printed by SeaLion. Best supported trees of each species-quartet of the clade combination CDOX are highlighted in blue dots and alternative supported trees highlighted in red dots. The majority of remaining species-quartets support for a rooted clade-quartet tree representing monophyletic Progoneata with Edafopoda (top right) while most rejected species-quartets support a best tree with Chilopoda and Diplopoda as monophyletic sister-group to Edafopoda (bottom left).

2.4.3 Clade-Quartet Related Tree Support

Median tree support for single clade-quartet relationships for the unfiltered and filtered ('RISK' and 'RISK+DIST') analysis of myriapod subgroup relationships with Edafopoda, inferred from species-quartets of the single clade-quartet 'CDOX' is shown in Tab. 2.10. Individual support values derived for each clade-quartet tree are standardized relative to each other, resulting in a support spectrum ranging from zero (indicating no support) to 1 (representing perfect support). The best supported clade-quartet trees with the respective counts of support underlying species-quartets are once more extracted in Table 2.11.

Table 2.10: Standardized median clade-quartet (4C) tree support with Edafopoda ('X') inferred from both unfiltered and filtered ('RISK' and 'RISK+DIST') species-quartet analyses, printed by SeaLion.

4C	Filter	4C Tree	Tree Code	Aggregate	Support
CDOX	unfiltered	(Out,(Chil,(Dipl,XPS)))	QT1	median	0.39
CDOX	unfiltered	(Out,(Dipl,(Chil,XPS)))	QT2	median	0.22
CDOX	unfiltered	(Out,(X,(Chil,Dipl)))	QT3	median	0.39
CDOX	RISK	(Out,(Chil,(Dipl,X)))	QT1	median	0.81
CDOX	RISK	(Out,(Dipl,(Chil,X)))	QT2	median	0.19
CDOX	RISK	(Out,(X,(Chil,Dipl)))	QT3	median	0.00
CDOX	RISK+DIST	(Out,(Chil,(Dipl,X)))	QT1	median	0.83
CDOX	RISK+DIST	(Out,(Dipl,(Chil,X)))	QT2	median	0.17
CDOX	RISK+DIST	(Out,(X,(Chil,Dipl)))	QT3	median	0.00

Table 2.11: Standardized median support of analysed species-quartets ('Nq') for the best clade-quartet ('4C') tree with Edafopoda ('X') in both unfiltered and filtered ('RISK' and 'RISK+DIST') quartet analyses, printed by SeaLion. The unfiltered analysis yielded low best-tree support for Chilopoda and Diplopoda as a monophyletic sister-group to Edafopoda ('QT3'). In contrast, both filtered analyses exhibited robust support for Progoneata with Edafopoda ('QT1').

4C	Filter	Best 4C Tree	Tree Code	Aggregate	Support	Nq
CDOX	unfiltered	(Out,(X,(Chil,Dipl)))	QT3	median	0.39	18560
CDOX	RISK	(Out,(Chil,(Dipl,X)))	QT1	median	0.81	5275
CDOX	RISK+DIST	(Out,(Chil,(Dipl,X)))	QT1	median	0.83	4676

2.4.4 Species Related Quartet Participations

A summarized overview of single species related quartet contributions in clade-quartet 'CDOX' of the unfiltered and filtered ('RISK', 'RISK+DIST') analyses are listed in Tab. 2.12. The

collective count of single species contributions, following species-quartet filtering, is depicted for the 'RISK' filter in Fig. 2.15 and for the 'DIST' filter in Fig. 2.16.

Table 2.12: Number of species participations in species-quartets of both unfiltered and filtered ('RISK' and 'RISK+DIST') clade-quartet (4C) analyses, printed by SeaLion.

OTU	Clan	4Clan	Unfiltered	RISK	RISK+DIST
Acerentomon_maius	O	CDOX	640	39	30
Acopauropus_ornatus	X	CDOX	4640	1495	1288
Anaspides_tasmaniae	O	CDOX	640	38	34
Anurida_maritima	O	CDOX	640	1	1
Araneus_diadematus	O	CDOX	640	212	165
Archegozetes_longisetosus	O	CDOX	640	1	0
Atelura_formicaria	O	CDOX	640	358	319
Callipus_foetidissimus	D	CDOX	1856	460	394
Clinopodes_flavidus	C	CDOX	1160	193	163
Craspedosoma_sp	D	CDOX	1856	502	475
Craterostigmus_tasmanianus	C	CDOX	1160	324	285
Cryptops_anomalans	C	CDOX	1160	260	233
Cryptops_hortensis_F2014	C	CDOX	1160	268	235
Damon_diadema	O	CDOX	640	614	551
Daphnia_pulex	O	CDOX	640	16	15
EDANI	O	CDOX	640	132	124
Egaenus_convexus	O	CDOX	640	480	407
Essigella_californica	O	CDOX	640	0	0
Eubranchipus_grubii	O	CDOX	640	11	10
Eudigraphis_takakuwai	D	CDOX	1856	802	733
Eupolybothrus_cavernicolus_sp	C	CDOX	1160	493	435
Eupolybothrus_cf_fasciatus	C	CDOX	1160	604	560
Eupolybothrus_tridentinus	C	CDOX	1160	498	442
Euscorpius_sicanus	O	CDOX	640	515	471
Glomeridella_minima	D	CDOX	1856	480	425
Hanseniella_nivea	X	CDOX	4640	1007	900
Hanseniella_sp	X	CDOX	4640	1511	1381
Haploglomeris_multistriata	D	CDOX	1856	511	450
Hemidiaptomus_amblyodon	O	CDOX	640	0	0
Henia_illyrica	C	CDOX	1160	187	162
Himantarium_gabrielis	C	CDOX	1160	256	232
Ixodes_scapularis	O	CDOX	640	49	49
LFUL	O	CDOX	640	277	255
Lithobius_forficatus	C	CDOX	1160	462	401
Machilis_hrabei	O	CDOX	640	313	268
Nebalia_bipes	O	CDOX	640	0	0
Nymphon_gracile	O	CDOX	640	218	210

Continues on next page(s).

Occasjapyx_japonicus_neu	O	CDOX	640	282	221
Ommatoiulus_sabulosus	D	CDOX	1856	489	408
Peripatoides_novaezealandiae	O	CDOX	640	425	411
Peripatopsis_capensis_F2014	O	CDOX	640	505	486
Periplaneta_americana	O	CDOX	640	352	275
Pogonognathellus_sp	O	CDOX	640	1	1
Polydesmus_complanatus	D	CDOX	1856	714	644
Polyxenus_lagurus	D	CDOX	1856	658	576
Polyzonium_germanicum	D	CDOX	1856	374	312
Schendyla_cf_carniolensis	C	CDOX	1160	201	173
Scolopendra_cingulata	C	CDOX	1160	370	324
Scolopocryptops_rubiginosus	C	CDOX	1160	311	278
Scutigera_coleoptrata	C	CDOX	1160	502	438
Strigamia_acuminata	C	CDOX	1160	182	163
Strigamia_maritima	C	CDOX	1160	164	152
Subilla_sp	O	CDOX	640	37	30
Symphylella_sp	X	CDOX	4640	1262	1107
Thalassisobates_littoralis	D	CDOX	1856	285	259
Tisbe_furcata	O	CDOX	640	0	0
Triops_cancriformis	O	CDOX	640	40	34
Vargula_hilgendorfii	O	CDOX	640	47	36
Xibalbanus_tulumensis	O	CDOX	640	312	273

End of table 2.12

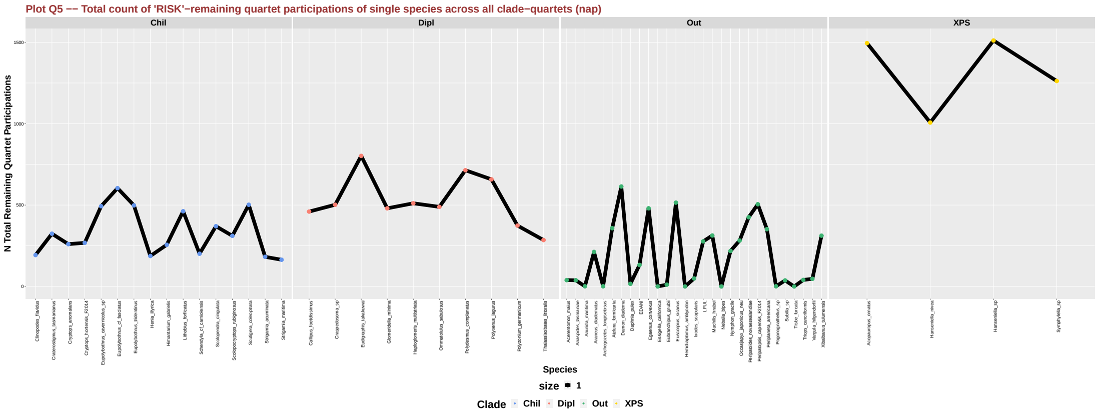

Figure 2.15: **'RISK' filtering**: Overall count of species (x-axis) related quartet participations (y-axis), remaining in clade-quartet with Edafopoda 'CDOX', printed by SeaLion.

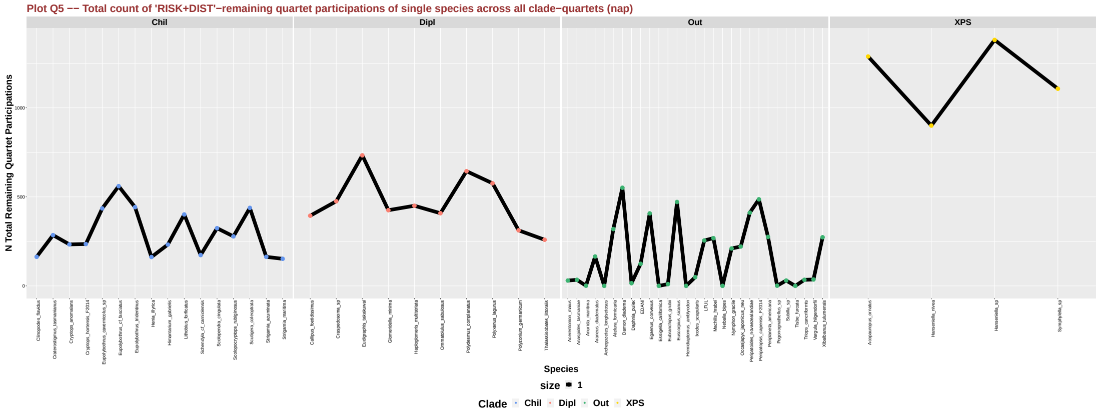

Figure 2.16: **'DIST' filtering**: Overall count of species (x-axis) related quartet participations (y-axis), remaining in clade-quartet with Edafopoda 'CDOX', printed by SeaLion.

2.4.5 Species Related Clade-Quartet Tree Support Contribution

The clade-quartet tree support contributions related to individual species are represented by the median tree support for each species involving clade-quartet, with tree support values normalized to each other. Unfiltered species-related support contributions for each clade-quartet tree are illustrated in Fig. 2.17. Species support contributions, following 'RISK' and 'RISK+DIST' species-quartet filtering analyses, are depicted as line plots in Fig. 2.18 and Fig. 2.19, respectively. Triangle plots illustrating species-related support contributions are presented in Fig. 2.20. The consistency of species-related best-tree support contributions to the tree of Szucsich *et al.* (2020) [5] with unfiltered and filtered quartets is demonstrated in Fig. 2.21.

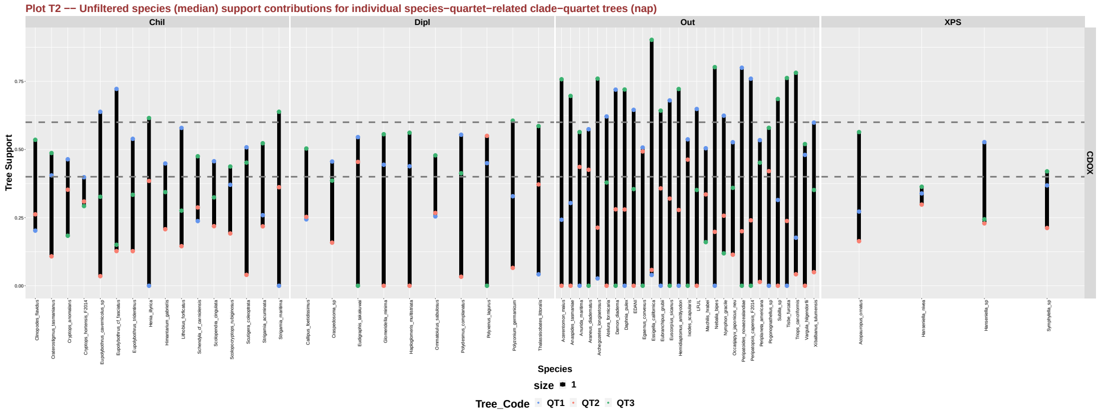

Figure 2.17: Support contributions from individual species (x-axis) in **unfiltered species-quartets** are depicted for each of the three trees (QT1, QT2, QT3; for corresponding newick string see Table 2.13) in clade-quartet 'CDOX' (y-axis, right), printed by SeaLion. Black vertical lines emphasize median support distances between different trees.

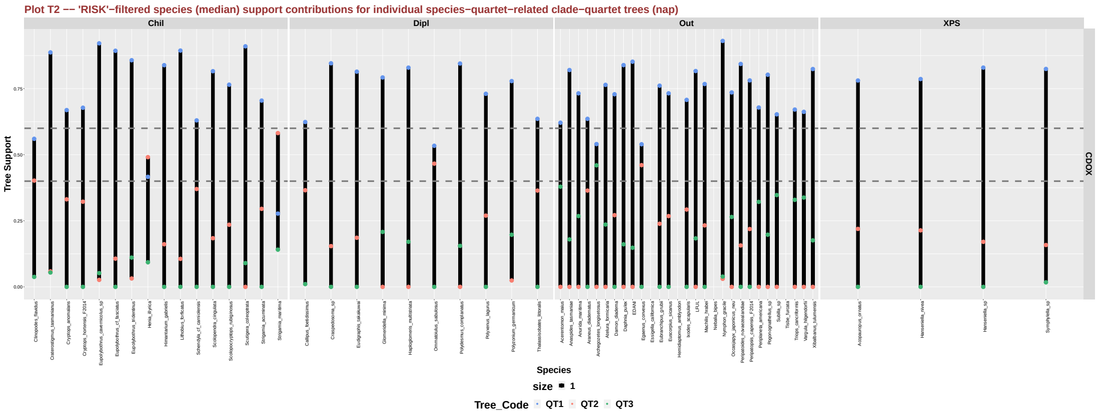

Figure 2.18: Support contributions from individual species (x-axis) in **'RISK' filtered species-quartets** are depicted for each of the three trees (QT1, QT2, QT3; for corresponding newick string see Table 2.13) in clade-quartet 'CDOX' (y-axis, right), printed by SeaLion. Black vertical lines emphasize median support distances between different trees.

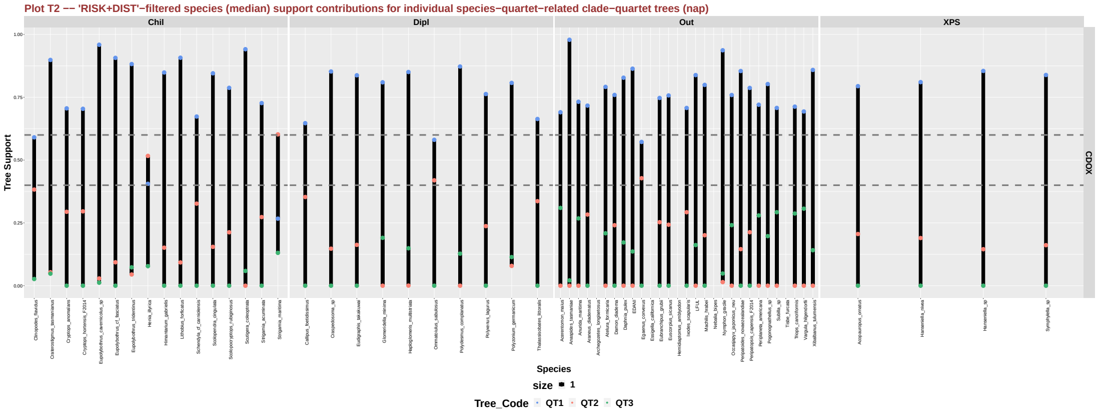

Figure 2.19: Support contributions from individual species (x-axis) in **'RISK+DIST' filtered species-quartets** are depicted for each of the three trees (QT1, QT2, QT3; for corresponding newick string see Table 2.13) in clade-quartet 'CDOX' (y-axis, right), printed by SeaLion. Black vertical lines emphasize median support distances between different trees.

Table 2.13: Designated tree codes, applicable to clade-quartet (4C) 'CDOX' with 'X' uniting Pauropoda and Symphyla.

**4C**	**QT1**	**QT2**	**QT3**
CDOX	(Out,(Chil,(Dipl,X)))	(Out,(Dipl,(Chil,X)))	(Out,(X,(Chil,Dipl)))

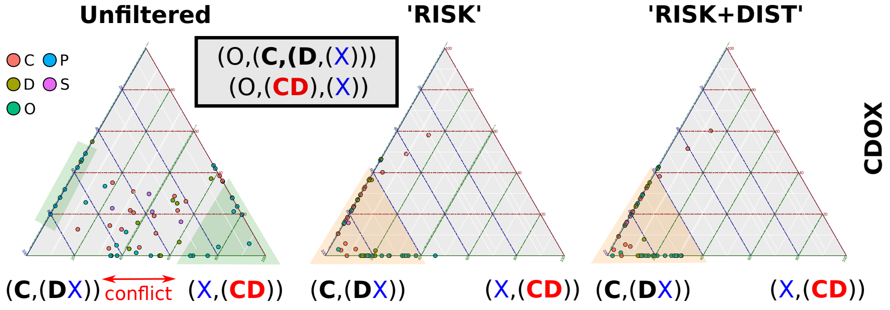

Figure 2.20: In the unfiltered data, there is outgroup conflicting support for monophyletic Chilopoda and Diplopoda, highlighted in green. The majority of filtered species-quartets show strong support for Chilopoda (C) as first ingroup lineage (highlighted in yellow), with 'X' representing Edafopoda. The inclusion of the 'DIST' filter marginally enhances the signal strength for this relationship.

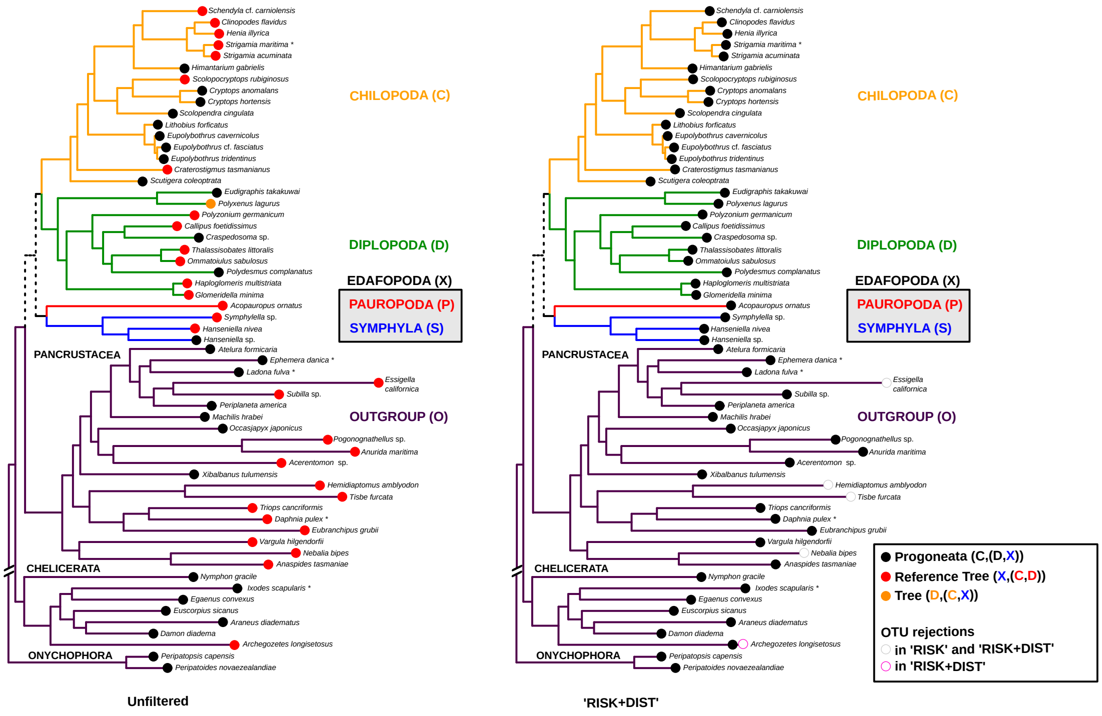

Figure 2.21: Comparison of the fit of species-quartet topologies to the reference tree (Szucsich *et al.* (2020) [5]) with unfiltered and filtered quartets. Black dots: species in topologies favouring the Progoneata; red dots: species in topologies favouring the clade Chilopoda + Diplopoda '(CD)' adjacent to Edafopoda (X). In the unfiltered data, there is a more or less balanced mixture of species with quartet participations supporting either tree, while species related best tree support contributions in filtered quartets are throughout consistent for Chilopoda (C) as first ingroup lineage (Progoneata).

2.4.6 Final Clade-Tree Support

Final clade-tree support for the unfiltered and filtered ('RISK', 'RISK+DIST') analyses of myriapod subgroup relationships with monophyletic Edafopoda are shown in Fig. 2.14.

Table 2.14: Support values for the three clade-trees in analyses with monophyletic Edafopoda, without and with quartet filters ('RISK' and 'RISK+DIST'). In the unfiltered approach, the best topology supports the monophyly of the clade Chilopoda + Diplopoda (labeled by '$\star$'). However, the diferences in support to the next best trees ($\Delta_{Best|2^{nd}}$, $\Delta_{Best|3^{rd}}$), especially to the topology with monophyletic Progoneata (highlighted in bold), the second-best tree, are close to zero, indicating a strong conflict between these toplogies. In contrast, in the filtered analyses the best tree support favoring Progoneata is significantly stronger (highlighted in bold) with larger support differences between the best and the other trees. Notably, the topology with the best unfiltered support receives zero support wih filtered data.

**Final Support**	**Best**	**2**nd	**3**rd	$\Delta_{Best\|2^{nd}}$	$\Delta_{Best\|3^{rd}}$
Unfiltered	$\star$ 0.79	**0.77**	0.44	0.02	0.35
'RISK'	**1.62**	0.38	$\star$0.00	1.24	1.62
'RISK+DIST'	**1.66**	0.34	$\star$0.00	1.32	1.66

CHAPTER 3

Simulations

3.1 Simulation Parameter

Nucleotide datasets oof the same alignment length as the original alignment are simulated with INDELible [6] using the original maximum-likelihood tree, including branch lengths, from Szucsich et al. (2020) [5]. An alternative tree assumes monophyletic Progoneata with Edafopoda. Branch lengths of the alternative tree relationship, as well as substitution model parameters for both tree simulations, are initially optimized with IQ-TREE [7] based on the original alignment. [5].

Table 3.1: INDELible parameter setting for simulation of ten character complete and ten character incopmplete data sets for the original and the alternative tree relationship ('testtree.tre') based on parameter assumptions estimated by IQ-TREE [7] multicore version 1.6.1 for Linux 64-bit (built Feb 16 2018) from the data published by Szucsich *et al*. [5]. Parameter highlighted in red are set off for character complete simulations.

**Parameter**	**Setting**
N data sets	10
TYPE	NUCLEOTIDE 1
indelmodel	LAV 2.4 400
indelrate	0.01747
newick	testtree.tre
output	FASTA
randomseed	12345 to 12354
rates	0.256 0.772 0
rootlength	95,797 bp
statefreq	0.285 0.199 0.355 0.161
submodel	GTR 3.41025 1.57644 1.0000 3.17315 6.76803

3.2 SeaLion Parameter Specifications

Our data simulations are analyzed using the same SeaLion parameters and clade definitions as described for our empirical analyses (see section 2.1).

3.3 Final Clade-Tree Support without Edafopoda

Final clade-tree support for the unfiltered and filtered ('RISK', 'RISK+DIST') analyses of myriapod subgroup relationships without monophyletic Edafopoda are shown in Fig. 3.2.

Table 3.2: Conclusive rooted clade-tree support obtained for the reference tree (Ref) featuring monophyletic Chilopoda and Edafopoda, and for Progoneata (Pro) across ten simulations using both character complete and incomplete (indel simulated) data (D). The simulations are based on either the reference tree or Progoneata. In all our analyses, the tree underlying the data consistently receives the strongest support, and this support is enhanced when species-quartets are filtered. Progoneata consistently ranks among the top three trees in simulations aligned with the reference tree, whereas the reference tree appears in the top three best trees only once in each character complete or incomplete simulation when the data is based on the Progoneata relationship.

Character Complete Data

	**Reference Tree Simulation**						**Progoneata Simulation**					
D	Unfiltered		'RISK'		'RISK+DIST'		Unfiltered		'RISK'		'RISK+DIST'	
	Ref	Pro	Ref	Pro	Ref	Pro	Ref	Pro	Ref	Pro	Ref	Pro
1	9.22	5.23	9.95	5.48	10.02	5.50	—	7.83	—	8.33	—	8.36
2	9.03	5.35	9.57	5.50	9.61	5.49	6.30	7.98	—	8.24	—	8.00
3	9.70	5.65	10.19	5.64	10.23	5.62	—	8.46	—	8.81	—	8.86
4	9.73	5.67	10.17	5.73	10.24	5.72	—	7.87	—	8.45	—	8.48
5	9.04	5.44	9.65	5.59	9.70	5.58	—	7.87	—	8.45	—	8.48
6	9.75	6.05	10.13	5.95	10.20	5.93	—	8.21	—	8.56	—	8.61
7	9.49	5.98	9.85	5.96	9.92	5.94	—	8.34	—	8.64	—	8.67
8	9.66	5.93	10.05	5.90	10.05	5.90	—	8.05	—	8.45	—	8.50
9	9.26	5.54	9.90	5.50	9.95	5.50	—	8.41	—	8.81	—	8.85
10	9.21	5.47	9.98	5.56	10.02	5.56	—	8.25	—	8.64	—	8.66

Character Incomplete Data

	**Reference Tree Simulation**						**Progoneata Simulation**					
D	Unfiltered		'RISK'		'RISK+DIST'		Unfiltered		'RISK'		'RISK+DIST'	
	Ref	Pro	Ref	Pro	Ref	Pro	Ref	Pro	Ref	Pro	Ref	Pro
1	9.37	5.91	9.97	5.86	10.03	5.85	—	8.22	—	8.67	—	8.70
2	9.87	6.11	10.49	6.07	10.54	6.05	—	8.66	—	8.88	—	8.95
3	9.62	5.80	10.10	5.80	10.16	5.79	—	7.68	—	8.20	—	8.74
4	9.78	6.62	10.45	6.34	10.49	6.32	6.63	7.56	6.33	8.04	—	8.08
5	9.98	5.88	10.43	5.89	10.50	5.88	—	7.77	—	8.28	—	8.32
6	8.64	5.77	9.41	5.82	9.47	5.81	—	8.42	—	8.79	—	8.84
7	9.35	5.39	9.96	5.48	9.99	5.48	—	8.51	—	8.76	—	8.83
8	9.44	5.82	10.23	5.85	10.23	5.85	—	8.51	—	8.76	—	8.80
9	8.97	5.74	9.59	5.75	9.62	5.74	—	8.17	—	8.81	—	8.62
10	10.07	6.09	10.52	5.98	10.56	5.98	—	8.16	—	8.65	—	8.67

3.4 Final Clade-Tree Support with Edafopoda

An example output of single species-quartet support for each of the three possible clade-trees of both reference tree simulated, with monophyletic Chilopoda + Diplopoda, and Progoneata simulated data is shown for the unfiltered and 'RISK+DIST' filtered analyses in Fig. 3.1. Final clade-tree support for the unfiltered and filtered ('RISK', 'RISK+DIST') analyses of myriapod subgroup relationships with monophyletic Edafopoda are shown in Fig. 3.3.

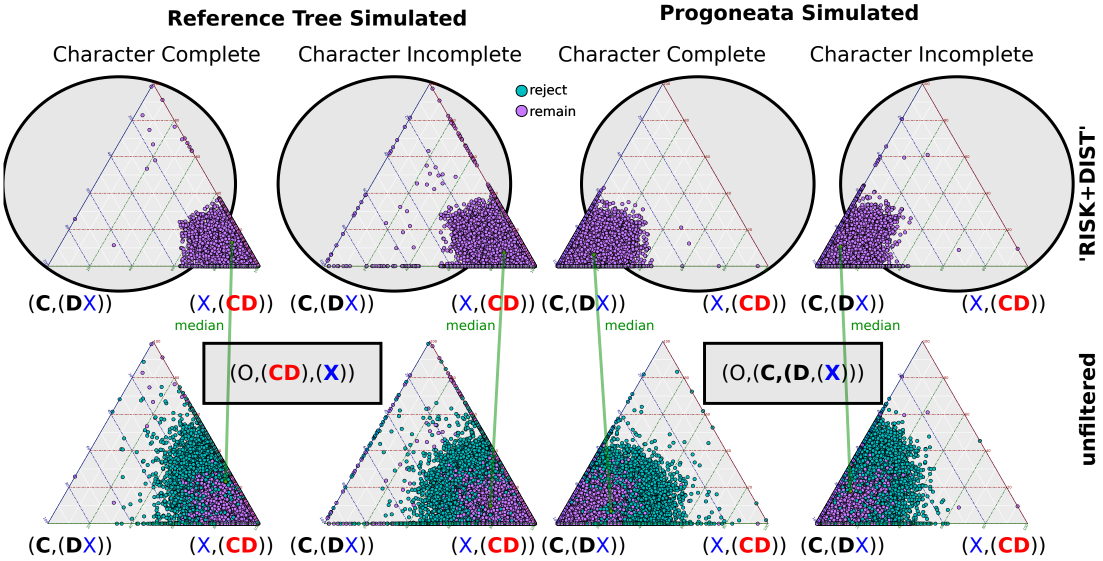

Figure 3.1: Representative illustration of analyzed species-quartet support from one of the ten Reference Tree simulations based on the tree of Szucsich *et al.* (2020) (left) with monophyletic Chilopoda and Diplopoda (CD, right triangle corner) or on Progoneata (right) with monophyletic Edafopoda 'X' (left triangle corner). Analyses of both tree simulations exhibit robust signal strength for tree-consistent relationships, with a slight signal improvement when species-quartets are filtered. Character incomplete (indel simulated) and character complete simulations yield quite similar results, with some additional conflicted quartet support identified in character incomplete simulations.

Table 3.3: Conclusive rooted clade-tree support obtained for the reference tree (Ref) featuring monophyletic Chilopoda and Edafopoda, and for Progoneata (Pro) across ten simulations using both character complete and incomplete data (D). The simulations are based on either the reference tree or Progoneata. In all our analyses, the tree underlying the data consistently receives the strongest support, and this support is enhanced when quartets are filtered.

Character Complete Data

	Reference Tree Simulation						Progoneata Simulation					
D	Unfiltered		'RISK'		'RISK+DIST'		Unfiltered		'RISK'		'RISK+DIST'	
	Ref	Pro	Ref	Pro	Ref	Pro	Ref	Pro	Ref	Pro	Ref	Pro
1	1.46	0.08	1.61	0.12	1.62	0.12	0.44	1.42	0.28	1.59	0.28	1.59
2	1.36	0.13	1.51	0.15	1.52	0.15	0.52	1.30	0.28	1.54	0.28	1.54
3	1.48	0.32	1.61	0.12	1.62	0.12	0.19	1.40	0.13	1.57	0.12	1.57
4	1.59	0.18	1.68	0.13	1.69	0.13	0.10	1.56	0.10	1.68	0.10	1.68
5	1.46	0.22	1.55	0.19	1.59	0.18	0.20	1.33	0.13	1.52	0.13	1.52
6	1.53	0.30	1.63	0.22	1.65	0.21	0.26	1.40	0.20	1.56	0.20	1.57
7	1.40	0.22	1.50	0.19	1.52	0.18	0.00	1.57	0.00	1.68	0.00	1.68
8	1.51	0.35	1.60	0.29	1.63	0.28	0.30	1.44	0.21	1.57	0.21	1.57
9	1.45	0.16	1.62	0.08	1.63	0.08	0.28	1.54	0.19	1.69	0.19	1.69
10	1.48	0.28	1.64	0.21	1.65	0.20	0.13	1.48	0.11	1.62	0.11	1.62

Character Incomplete Data

	Reference Tree Simulation						Progoneata Simulation					
D	Unfiltered		'RISK'		'RISK+DIST'		Unfiltered		'RISK'		'RISK+DIST'	
	Ref	Pro	Ref	Pro	Ref	Pro	Ref	Pro	Ref	Pro	Ref	Pro
1	1.42	0.28	1.64	0.20	1.64	0.20	0.14	1.50	0.12	1.67	0.12	1.67
2	1.48	0.33	1.63	0.22	1.63	0.22	0.27	1.55	0.20	1.64	0.20	1.64
3	1.53	0.31	1.64	0.24	1.64	0.24	0.20	1.37	0.16	1.53	0.16	1.53
4	1.48	0.52	1.67	0.33	1.67	0.33	0.57	1.21	0.43	1.38	0.18	1.39
5	1.59	0.19	1.68	0.17	1.68	0.17	0.26	1.29	0.18	1.46	0.18	1.47
6	1.37	0.38	1.52	0.26	1.52	0.26	0.07	1.44	0.04	1.61	0.04	1.61
7	1.49	0.00	1.62	0.05	1.62	0.05	0.24	1.51	0.18	1.61	0.18	1.61
8	1.52	0.29	1.68	0.21	1.68	0.21	0.26	1.52	0.17	1.65	0.17	1.65
9	1.41	0.34	1.56	0.25	1.56	0.25	0.35	1.43	0.23	1.59	0.23	1.60
10	1.54	0.24	1.68	0.16	1.68	0.16	0.27	1.43	0.17	1.63	0.17	1.63

Bibliography

[1] Kück P, Wilkinson M, GroßC, Foster PG, Wägele JW (2017) Can quartet analyses combining maximum likelihood estimation and hennigian logic overcome long branch attraction in phylogenomic sequence data? PLoS ONE 12: e0183393. (Cited on pages 1, 5, 6 and 9.)

[2] Kück P, Longo GC (2014) FASconCAT-G: extensive functions for multiple sequence alignment preparations concerning phylogenetic studies. Front Zool 11: 81. (Cited on page 3.)

[3] Misof B, Meyer B, von Reumont BM, Kück P, Misof K, et al. (2013) Selecting informative subsets of sparse supermatrices increases the chance to find correct trees. Bmc Bioinformatics 14: 1–13. (Cited on pages 7 and 8.)

[4] Foster PG (2004) Modeling compositional heterogeneity. Syst Biol 53: 485-495. (Cited on page 9.)

[5] Szucsich NU, Bartel D, Blanke A, Böhm A, Donath A, et al. (2020) Four myriapod relatives – but who are sisters? No end to debates on relationships among the four major myriapod subgroups. BMC Evol Biol 20: 144. (Cited on pages 9, 10, 30, 32 and 35.)

[6] Fletcher W, Yang Z (2009) INDELible: A flexible simulator of biological sequence evolution. Mol Biol Evol 26: 1879-1888. (Cited on page 35.)

[7] Nguyen LT, Schmidt HA, Haeseler A, Minh BQ (2015) Iq-tree: A fast and effective stochastic algorithm for estimating maximum-likelihood phylogenies. Mol Biol Evol 32: 268-274. (Cited on page 35.)
